# Linear Dichroism Microscopy Resolves Competing Structural Models of a Synthetic Light-Harvesting Complex

**DOI:** 10.1021/jacs.4c17708

**Published:** 2025-02-04

**Authors:** Alexey V. Kuevda, Mónica K. Espinoza Cangahuala, Richard Hildner, Thomas L. C. Jansen, Maxim S. Pshenichnikov

**Affiliations:** Zernike Institute for Advanced Materials, University of Groningen, Nijenborgh 3, 9747 AG Groningen, The Netherlands

## Abstract

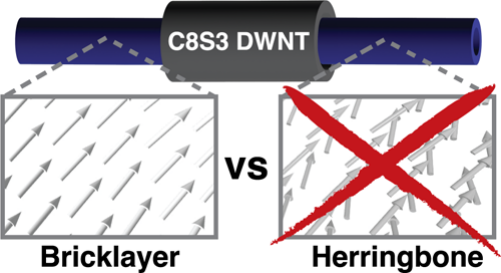

The initial stages of photosynthesis in light-harvesting antennae, driven by excitonic energy transport, have inspired the design of artificial light-harvesting complexes. Double-walled nanotubes (DWNTs) formed from the cyanine dye C8S3 provide a robust, self-assembled system that mimics natural chlorosomes in both structure and optical properties. Two competing molecular packing models—bricklayer (BL) and herringbone (HB)—have been proposed to explain the structural and optical characteristics of these DWNTs. This study resolves the debate by combining theoretical analysis with advanced polarization-resolved wide-field photoluminescence microscopy. Quantum-classical simulations reveal reduced linear dichroism (LDr) as a decisive parameter for distinguishing between the models. Experimental measurements of single DWNTs yielded LDr values as high as 0.93, strongly favoring the BL model. The BL model’s unique excitonic patterns, dominated by negative couplings among individual chromophores, generate superradiant exciton states with transition dipoles preferentially aligned along the nanotube axis. In contrast, the HB model’s mixed positive and negative couplings produce destructive interference, leading to a weaker alignment of transition dipoles. Our approach deepens the understanding of the structure–property relationships in self-assembled systems and demonstrates the potential of slip-stacking engineering to fine-tune excitonic properties for artificial light-harvesting applications.

## Introduction

**Figure 1:**
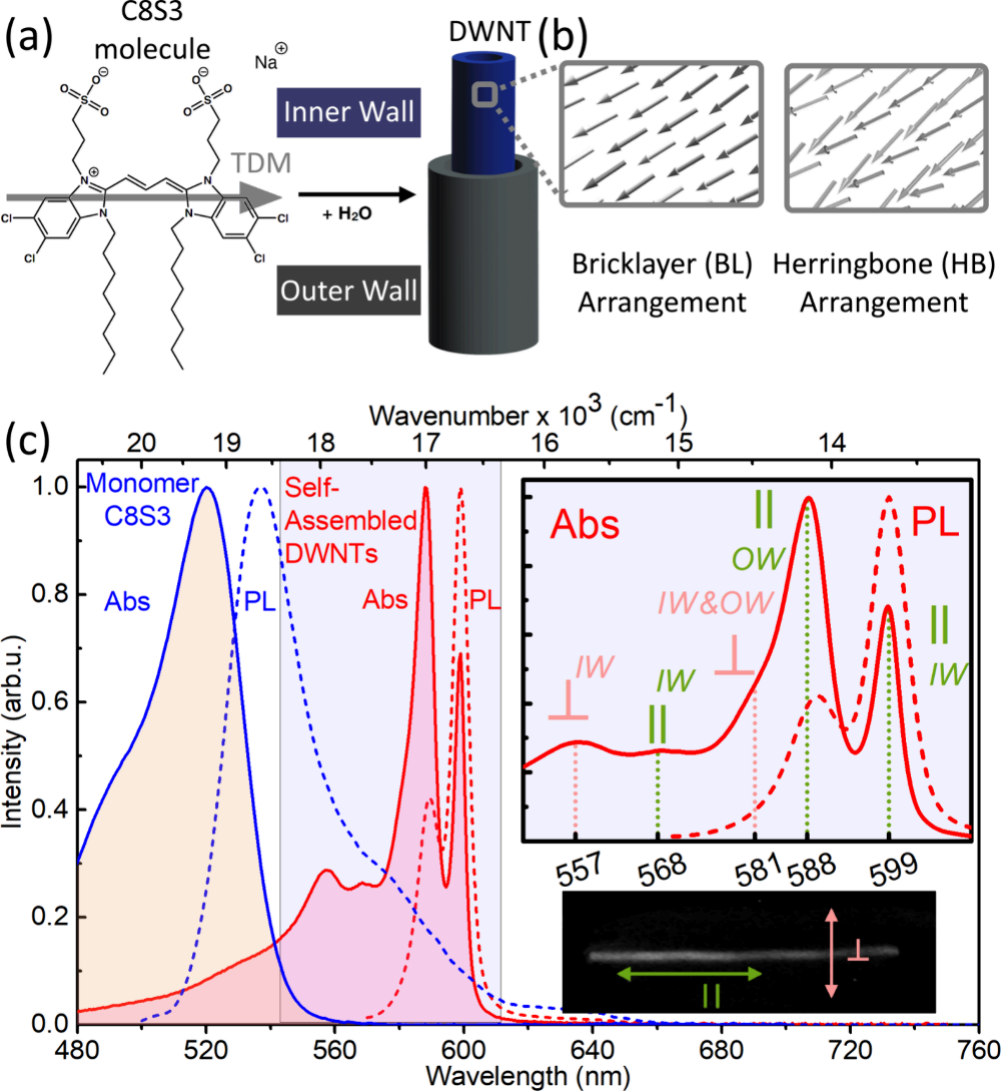
Basic properties of double-walled nanotubes (DWNTs). (a) Chemical structure of the monomer C8S3 molecule and schematic of a DWNT. The gray arrow shows the direction of the transition dipole moment (TDM). (b) Schematic orientations of monomer dipole moments for the bricklayer (left) and herringbone (right) molecular packing as seen from inside the DWNTs. The transition dipole moments are represented by arrows originating from the center of each C8S3 molecule. For more detailed structures, see SI, Figure S1. (c) Absorption (solid curves) and PL (dashed curves) spectra of the C8S3 monomers dissolved in methanol (blue) and DWNTs in water (red). The upper inset shows the polarization assignments for the absorption peaks: ∥ represents polarization along the long axis of the DWNT, while ⊥ indicates polarization perpendicular to it. IW and OW stand for the inner and outer walls, respectively. The lower inset displays a PL image of a DWNT, with the two polarization directions marked.

The initial stages of photosynthesis occur in light-harvesting antennae, which absorb sunlight and efficiently transport energy^1,2^ as delocalized excited states, or excitons, through a network of tightly packed pigments to the reaction center.^3^ The remarkable efficiency of excitonic transport in natural photosynthetic light-harvesting antennae,^4−6^ such as the chlorosomes found in green sulfur bacteria, has inspired efforts to learn from these systems and replicate their features under laboratory conditions.^7^ Double-walled nanotubes (DWNTs) formed from the C8S3 cyanine dye^8,9^ (Figure 1a), featuring a central functional core with hydrophilic and hydrophobic chains, exhibit a striking structural resemblance to natural chlorosomes.^10−12^ These DWNTs consist of two concentric tubular structures nested within one another (inner and outer nanotubes) with diameters of ∼6 and ∼14 nm, respectively, and lengths extending to several microns. DWNTs are self-assembled in an aqueous environment as a result of the delicate interplay between van der Waals forces, halogen/hydrogen bonds, and interlocking sulfonate groups.^13−15^ Structurally simple and remarkably homogeneous, yet closely resembling natural light-harvesting antennas, C8S3-based DWNTs have been extensively studied and modeled over the past three decades.^9,16−19^

The absorption spectrum of methanol-diluted C8S3 molecules is typical of a cyanine dye: it displays a very broad absorption centered around 520 nm with a vibronic progression (Figure 1c). Upon self-assembly, the absorption spectrum undergoes a dramatic transformation: two sharp, intense peaks emerge at 598 and 588 nm, accompanied by a weaker and broader shoulder at higher energy.^20,21^ These spectral features are attributed to the formation of multiple excitonic states due to strong intermolecular coupling within each nanotube (NT) of the DWNT structure.^16,22,23^ It has been shown^20,24^ that the main 598 and 588 nm peaks arise from exciton absorption in the inner and outer nanotubes, respectively, while the high-energy shoulder originates from higher energy exciton absorption of both nanotubes.^21,25^ The photoluminescence (PL) spectrum of DWNTs also exhibits characteristic excitonic features: narrow emission lines from the lowest-energy exciton states and the virtual absence of a Stokes shift (Figure 1c, red dashed lines).

Next to the absorption and PL data, linear dichroism (LD) of DWNT excitons, i.e. the difference in absorption or PL of orthogonally polarized light is proven to be highly valuable for linking structural and excitonic^26^ properties:
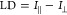
1where *I*_∥_ and *I*_⊥_ are the intensities of the transmitted light (or PL) in two orthogonal directions. To make LD independent of specific experimental conditions and enable comparisons across different settings, LD is conventionally normalized by the sum of the two intensities, resulting in the dimensionless reduced linear dichroism LDr:
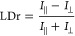
2In essence, LDr reveals to what extent the photons absorbed or emitted have a well-defined polarization. It was determined experimentally^27−29^ that the lowest-energy (and thus strongest) transition in each nanotube’s absorption spectrum is preferentially polarized along the DWNT’s long axis, while transitions at the higher-energies are polarized either parallel or perpendicular to it (Figure 1c, inset).

Over the past two decades, significant efforts have been devoted to understanding and explaining the optical properties of DWNTs. In 2004, a simple *bricklayer* (BL) model with one molecule per unit cell was proposed to reproduce the overall absorption and LD spectra of the DWNT system.^16^ The term *bricklayer* references a model in which the molecules are arranged in a systematic, layered fashion, similar to how bricks are laid in construction (Figure 1b). The slip in the two walls was slightly different, while all other unit cell parameters (brick size) were identical for both walls. However, a few years later, when the absorption spectrum of the isolated inner wall was experimentally separated using redox chemistry,^30^ its four peak structure was revealed. This result could not be explained by the BL model, which assumed one molecule per unit cell and produced only two peaks for each wall. In response, the two-molecule per unit cell herringbone (HB) model was proposed,^30^ in which the molecular arrangement is reminiscent of the skeleton of a herring, with alternating diagonal elements creating a zigzag pattern (Figure 1b). The HB model successfully reproduced the experimentally observed four-peak structure of the inner wall absorption spectrum as well as the LD spectrum. The herringbone angle between the two molecules in the unit cell was crucial for revealing both Davydov components in the spectra.

In 2015, a molecular dynamics model of the DWNT was developed.^31^ It used a BL structure with two molecules per unit cell where an energy difference between the two molecules was used to introduce the Davydov splitting. The predicted spectra successfully matched the experimental data of the full DWNT, when a weak electronic coupling between the chromophores but a strong vibronic coupling was assumed. We note in passing that this assumption is inconsistent with large electronic couplings, which were estimated using the known transition dipole of the cyanine dye.^32^ Next, a multiscale model^32^ was developed combining molecular dynamics, density functional theory (DFT), dimer X-ray structures,^33^ and optical spectroscopy. Modeling in an iterative procedure yielded an atomistic HB model, with two molecules per unit cell. This approach once again enabled a consistent description of the DWNT absorption spectra.

Both the original BL and HB models relied on cryo-TEM data for structural constraints such as diameters of inner and outer walls, while the unit cell arrangement and molecular packing were determined via fitting of optical spectra. This approach was recently challenged by Caram and co-workers, who conducted high-resolution cryogenic transmission electron microscopy (cryo-TEM) experiments to directly resolve the molecular structure of DWNTs.^34^ Their findings revealed that all the molecules in the inner wall (IW) are in a BL arrangement, and that a six-molecule unit cell forms the basis for the IW structure with a C_5_-rotational symmetry around the tube axis. It was also suggested that the interlocking sulfonates are responsible for the slip-stacked packing geometry, which is essential for the DWNT optical properties such as the spectral red-shift and spectral narrowing. Based on this structure, a simplified dimer-type exciton model was proposed resulting in optical absorption and LD spectra of the IW in line with experimental observations. The simplified dimer model may, however, not account for all details of the full hexamer molecular structure, calling for the use of the full detail of the structural model. The detailed structure of the outer wall (OW) could not be determined, but it was suggested that it is formed by helically wound strips separated by gaps resulting in an overall C_5_-rotational symmetry around the tube axis.

As a result of these efforts, two competing models emerged: the HB model and the BL model, revised to accommodate a larger unit cell. Both models accurately describe the absorption and LD spectra of the inner wall at a *qualitative* level, yet the applicability of each model remains under continued scrutiny. Hence, currently the critical challenge lies in devising effective techniques for distinguishing between the two. For the theoretical aspect, realistic calculations of optical properties must be performed that treat both structural models on an equal footing (e.g., on the full molecular level), resulting in *quantitative* (rather than the previously qualitative) parameters to predict a measurable difference in optical spectroscopy. Similarly, optical experiments must be designed in such a way to allow a direct comparison with theoretical predictions.

Here, we combine theoretical analysis with polarization-resolved wide-field PL microscopy of individual DWNTs to distinguish between the two structural models. Molecular-level exciton models are employed to perform quantum-classical spectral simulations of the inner wall structures. The theoretical predictions indicate that LDr can decisively differentiate between the models. For the two models, we observed a completely different nature of the emitting exciton states, arising from unique coupling patterns in the stacking geometry of the two structures. Experimentally, we observe LDr values that clearly favor the BL structure. The approach developed herein not only enables the discrimination of specific DWNT structures but also advances the use of optical spectroscopy in combination with theoretical modeling in distinguishing among various structural models of other self-assembled supramolecular systems.

## Results and Discussion

**Figure 2:**
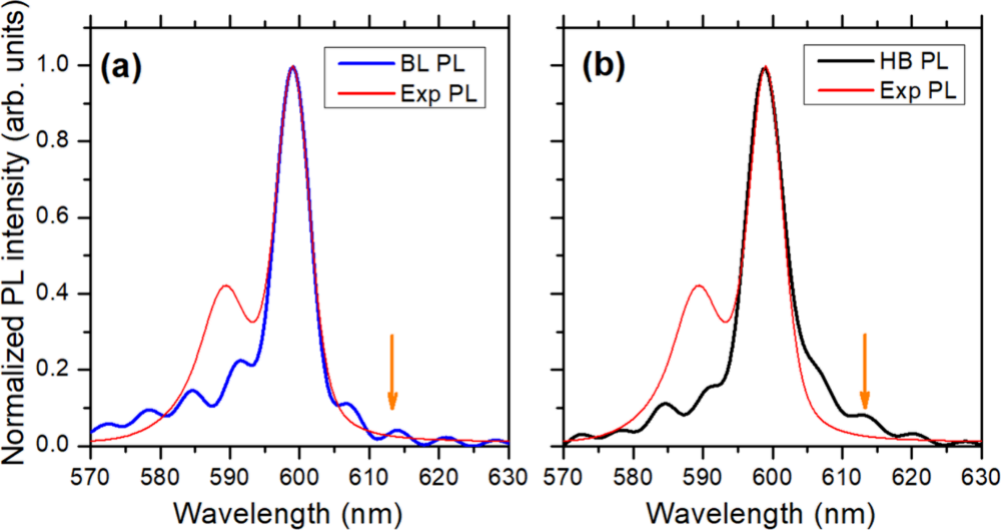
Calculated PL spectra of the inner wall for the bricklayer (a) and herringbone (b) models. For comparison, the experimental PL spectrum, that comprises PL from both inner and outer walls, is shown in red. The 590 nm peak originating from the outer wall’s PL, was not included in the modeling. The calculated spectra were shifted by −12.5 nm (a) and −11.4 nm to provide the best match with the PL spectrum of the inner tube. The orange arrows indicate the end wavelength (613 nm) for LDr calculations.

As a reporter of experimental observables, we focus on DWNT PL because it arises exclusively from the lowest-energy exciton transitions, eliminating complications associated with the spectral overlap of transitions with different orientations as seen e.g. in absorption (Figure 1c, inset). Figure 2 shows the PL spectra obtained for the two structural models of the IW using eq S3, along with the experimental PL spectrum of the full DWNT. The peak width for both structural models matches the experiment very well suggesting that the disorder model derived from the multiscale modeling^32^ is appropriate. In the red wing the BL model signal appears to have a longer tail that matches the experimental data slightly better than the HB model. The subpeak structure arises from ringing in the Fourier transform of the calculated response function. Overall, the PL data slightly favors the BL model; however, the difference is too small to draw any definitive conclusions.

Next, we examine the LDr which is recast from eq 2 as
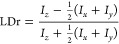
3where *I*_*i*_ (*i* = *x*, *y*, *z*) stand for PL emitted into the *i*-direction, the *z* axis coincides with the DWNT long axis, and the PL intensity into the orthogonal direction is evenly divided between the *x* and *y* components. A value of zero indicates no preferential polarization, which may result from either an isotropic distribution of transition dipoles of the emitting exciton states or a structured distribution resulting in equal emission from transition dipoles parallel with and perpendicular to the DWNT axis. A value of one arises when all emitting transition-dipole moments are parallel with the DWNT axis, and a value of minus one results when all emitting transition dipoles are perpendicular to the DWNT axis. For both the BL and HB structures the angles of the molecular transition-dipole moments are such that LDr values distributed around zero would emerge if the molecules were not strongly coupled.

**Figure 3:**
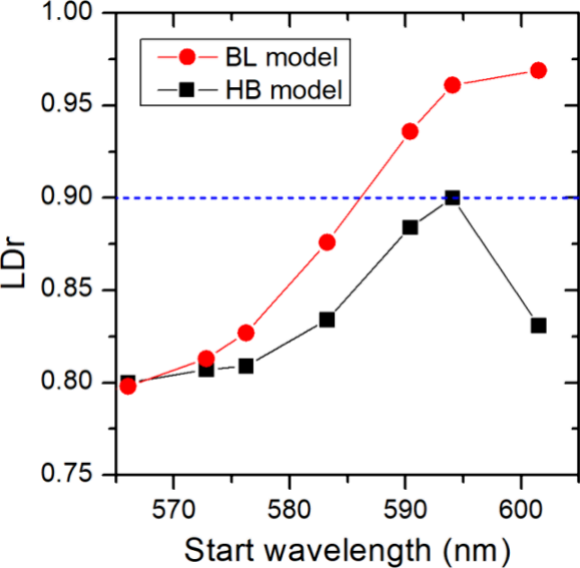
LDr calculated after averaging the simulated, spectrally shifted polarized PL signal in Figure 2 in the range from the start wavelength shown in the graph up to 613 nm. Red dots and black squares stand for LDr obtained for BL and HB models, respectively. Each dot represents a separate calculation. The blue dashed line indicates the minimum LDr value required to effectively differentiate between the BL and HB models.

To determine the LDr for the two models, we integrated the PL signal between 613 nm and different lower wavelengths between 565 and 605 nm before calculating the LDr according to eq 3 (for details, see SI, Section S7). The result is shown in Figure 3 for the two IW models. For both the BL and HB models the LDr is the largest at the main peak and at the red tail of the PL band, which is known^35^ to be dominated by exciton states predominantly polarized along the DWNT axis, while the LDr decreases to ∼0.8, when including states on the blue side of the main peak (<590 nm), where the exciton states polarized perpendicularly to the main axis contribute as well.

Comparing the BL and HB models, the difference in the LDr becomes quite substantial when the spectral integration window is reduced, and the BL model predicts a significantly larger LDr value for the spectral region of the IW PL. In the theoretical models, the DWNT axis is perfectly defined, and one will always expect a higher degree of alignment than in experiments. This, thus, suggests that if the experimental LDr value is above the largest value found for the HB model of ∼0.9 (blue dashed line in Figure 3), we can rule out this model leaving the BL model as the only feasible model for the IW.

Discriminating LDr values at the level of 0.9 presents a significant experimental challenge. Most experimental data on LD have been derived from bulk DWNT measurements,^27,36^ which rely on the streaming flow technique, i.e. the alignment of DWNTs along the direction of solution flow in a cell to create net anisotropy.^27−29,37,38^ An alternative approach involves using Mueller polarimetry to analyze the local regions that exhibit spontaneous orientation following the drying of the solution on a surface.^39,40^ However, imperfect alignment of the DWNTs causes a distribution of their orientations, and inherently results in strongly underestimated LD values.^27^ This constraint can be overcome in single-object microscopy with polarization-resolved PL detection^41−43^ where PL from each individual DWNT is analyzed. This approach on DWNTs was pioneered by Eisele et al.^44^ who utilized near-field scanning optical microscopy to report LDr values as high as 0.67. However, the values reported may have been affected by the metal-coated fiber tip in close proximity to the sample. Imperfections of the coating can give rise to spurious background light and distort polarization-sensitive measurements.^45,46^ Furthermore, out-of-focus unpolarized background PL, which is likely caused by the high concentration of DWNTs in the sample, may have also contributed to the decrease in LDr values.

Taking all these factors into account, we opted for a single-DWNT all-optical microscopy setup with separate excitation and detection channels to allow for independent polarization control of both the excitation radiation and the detected PL (see the SI, Section S3). Such dual-objective geometry enhances the LD resolution of the setup,^47−49^ which is typically limited in single-objective schemes by (de)polarization caused by the dichroic beamsplitter separating the excitation and PL collection channels.^50,51^ It also eliminates the need for sample rotation, which requires precise alignment of a DWNT under investigation with the microscope’s optical axis.^43^ A rotating half-wave plate and a Wollaston prism were positioned in the PL detection channel in front of the CMOS camera to sample all PL polarizations and spatially separate the two orthogonally polarized PL images.^52,53^ The out-of-focus unpolarized contribution to the PL signal was minimized by using diluted samples with effectively a monolayer of spatially isolated DWNTs immobilized in a sugar matrix (SI, Section S6).

**Figure 4:**
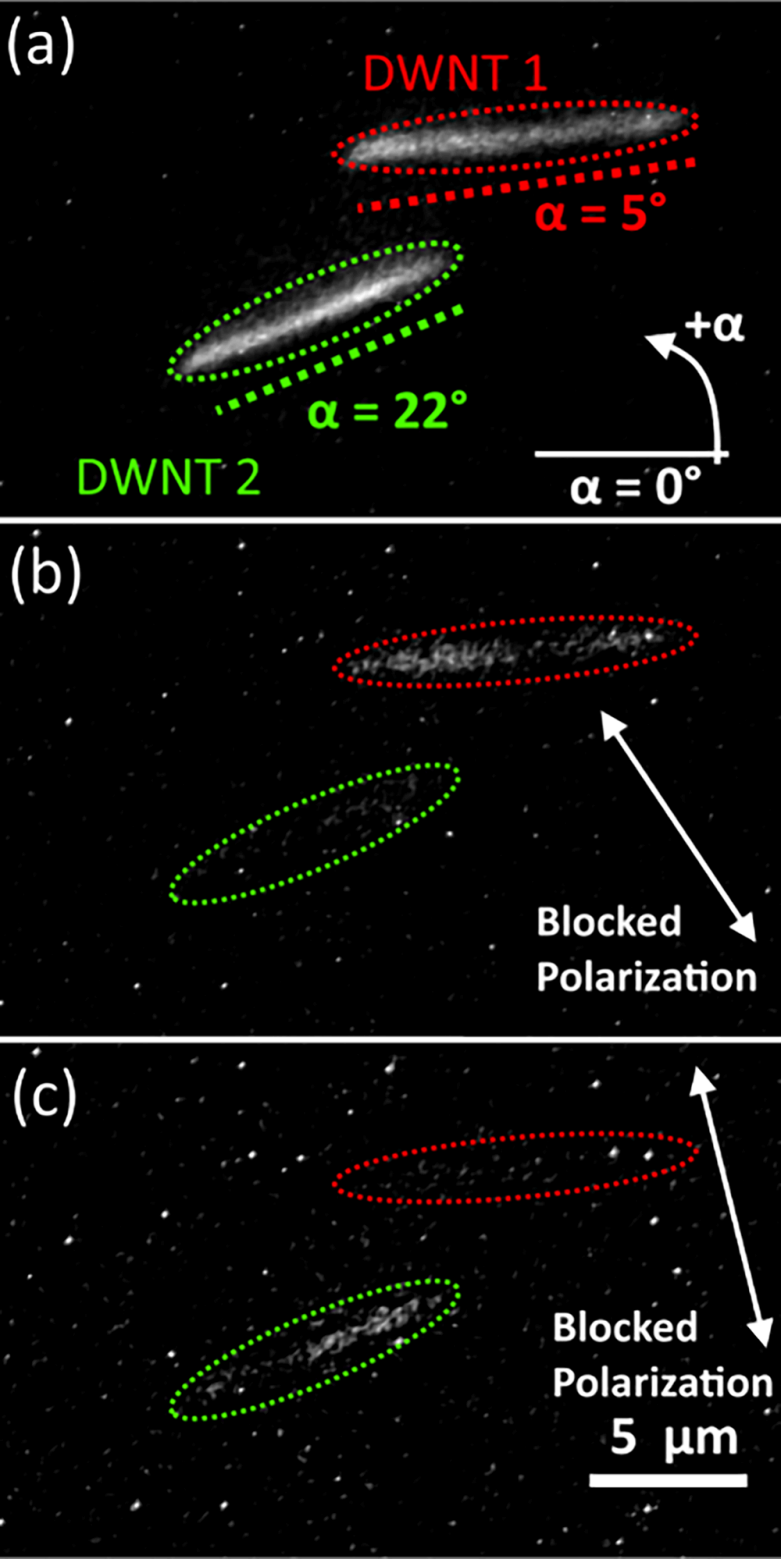
Polarization-resolved PL microscopic imaging. (a) Isotropic PL image of two DWNTs (highlighted by red and green dashed ovals). Straight dashed lines and corresponding numbers (in red and green) represent the geometrical orientations of the DWNTs. The zero-degree angle is indicated by the solid white line, with angles measured counterclockwise. (b, c) Polarized PL images of the same area with the blocked polarization direction indicated by the white arrow. To enhance the visibility of DWNTs in blocked polarizations, the brightness was increased by a factor of 3 (b) and 5 (c) compared to the image in (a).

**Figure 5:**
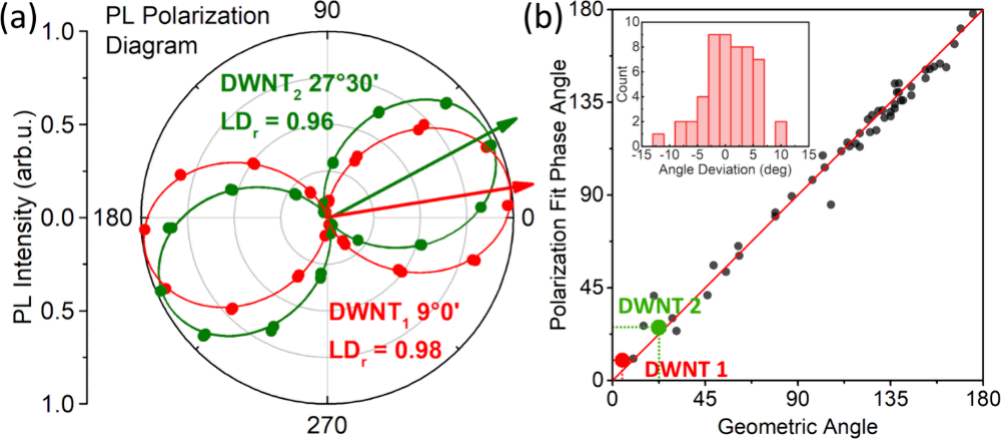
Analysis of the polarization-resolved PL images. (a) PL polarization diagram from the two DWNTs in Figure 4. Colors of the diagrams correspond to the highlighted colors of the respective DWNTs in Figure 4. The angles shown represent phase shifts extracted from sine-squared fits (solid lines) to the experimental PL intensity data (dots) at different half-wave plate orientations (for the fits, see Figure S9). Reduced Linear Dichroism (LDr) values are also provided for each DWNT. (b) Correlation plot comparing the geometrical orientations of the DWNTs, as extracted from isotropic PL images, with the polarization angle. The red diagonal line represents perfect correlation. The inset shows a histogram of the deviation between polarization and geometrical angles. Colored dots show data on two DWNTs from Figure 4.

Figure 4a shows an isotropic image of two close-by DWNTs. Image analysis reveals their orientations to be 5 and 22° counterclockwise relative to the horizontal axis. Figure 4a,b display polarization-resolved images at half-wave plate angles of 52 and 62°, respectively, which are close to the angles where the PL from either DWNT is almost completely suppressed (SI, Figure S9). This indicates a close match between the geometrical and polarization angles, i.e., the DWNT PL is mostly polarized along the long axis. This is further demonstrated in Figure 5a, which shows the complete PL polarization diagram. Fitting the PL data to a sine-squared function yields the refined phase shift, providing the orientation of the exciton transition dipole moment with an average uncertainty of ∼1° (SI, Figure S8).

This procedure of determining geometrical and polarization angles was repeated for 54 separate linear DWNTs or DWNT segments, with the results presented as a correlation plot in Figure 5b. The experimental points cluster closely around the diagonal (red line) with a Pearson correlation coefficient of *R* = 0.99. Deviations from perfect correlation make a narrow distribution around zero angle (Figure 5b, inset) which could be compared with similar distributions derived for the excitons in the BL and HB models (SI, Figure S14). The HB model yields a much wider angle distribution, which is hardly compatible with the experimental data. In contrast, the BL model predicts a closer match, although the experimental distribution is slightly broader which may be attributed to imperfect planar orientations of the DWNTs and accumulated experimental uncertainties. These differences in the distributions arise from the fundamentally distinct nature of the exciton wave functions in the two structures (vide infra).

In the previous microscopy study,^44^ the implicit assumption was made that the PL transition dipole moments were oriented strictly along the DWNT long axis; however, this assumption was not experimentally verified. As we mentioned earlier, disorder within the DWNT can lead to exciton localization in certain sections, potentially disrupting the cylindrical symmetry^16,19,54^ and resulting in exciton dipole moments with different alignments. Figure 5b provides direct experimental evidence that the exciton PL transition dipole moments are primarily aligned along the long axis of the DWNT.

**Figure 6:**
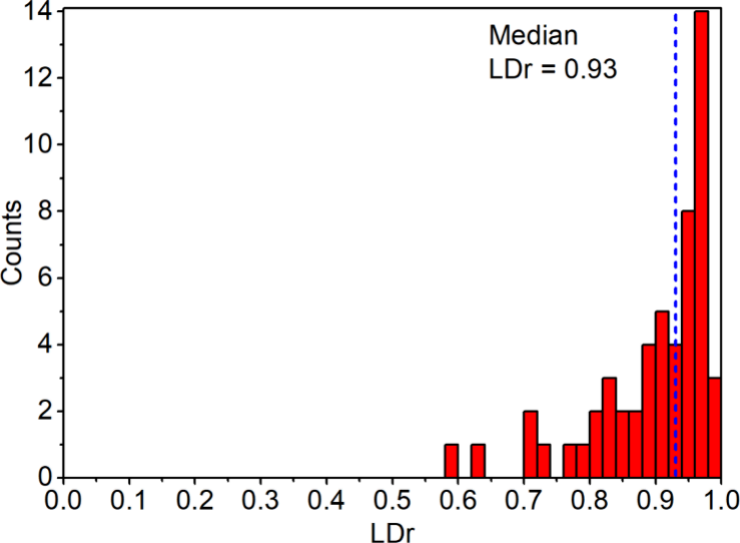
Histogram of the experimental LDr values calculated for 54 DWNTs. The experimental LDr values (eq S5) were defined identically to eq 2. The bin size is 0.02.

Figure 6 shows the distribution of LDr values measured from 54 DWNTs, with a median value of 0.93 and the most probable value of 0.97 which is significantly higher than the LDr = 0.67 reported earlier.^44^ In Figure S13 of the SI the analysis of the LDr distribution for the exciton states is given for the two models, demonstrating a sharper distribution for the BL model than for the HB model. It is important to note that the simulated LDr distributions represent individual exciton states, including a few with negative LDr values, corresponding to transition dipoles oriented perpendicular to the tube axis. In contrast, the experimental measurements yield exciton-averaged LDr values, which naturally do not approach values close to ±1. Imperfections in the samples–such as nonplanar orientation or unwanted residual background–can also contribute to reduced LDr values. These factors enhance the left tail of the experimental distribution, resulting in a lower median LDr value. Therefore, the observed median value of 0.93—as high as it is—most likely represents the lower estimate of the true distribution. Interestingly, despite being soft, highly dynamic supramolecular structures, DWNTs along with other J-aggregated tubular assemblies,^43^ exhibit remarkably strong linear dichroism, which is typically characteristic of solid-state crystalline systems.^55,56^

A careful examination of the DWNT images (Figure 4a) reveals that the PL intensity is not entirely uniform along their lengths (for data analysis, see Supporting Information, Section S8). Intensity variations on the scale of a few micrometers could be attributed to the distribution of the excitation intensity profile with a size of approximately 10 μm which is about the DWNT length (Figure S15a). Variations on shorter length scales (Figure S15b) may arise from dislocations or defects in the DWNT segments formed during DWNT growth, which affects either absorption at higher excitonic states, or PL quantum yield, or both. Despite these intensity variations, the LDr values calculated along the DWNT length (Figure S15) remain constant and are consistent with the average values reported in Figure 4.

One could also consider that the samples may contain either a mixture of DWNTs with HB and BL packing or DWNTs with domains of each structure. If the sample were a mixture, the observed angular distribution would represent a weighted sum of the distributions from each structure. Based on experimental observations, a significant contribution from HB DWNTs can be excluded. If the two structures were intermixed within the same DWNTs, the outcome would depend on the domain size. For domains larger than the exciton delocalization size, the result would be the same as the mixed situation as described above. If the domain size were smaller or comparable to the exciton delocalization size, it would lead to inhomogeneous broadening of the absorption and fluorescence spectra, an effect not observed in 2D correlation spectra.^24^ Furthermore, the angular distribution could become even broader than predicted by the HB model, as the inhomogeneous disorder would cause exciton localization due to mismatches between the BL and HB lattices.

**Figure 7:**
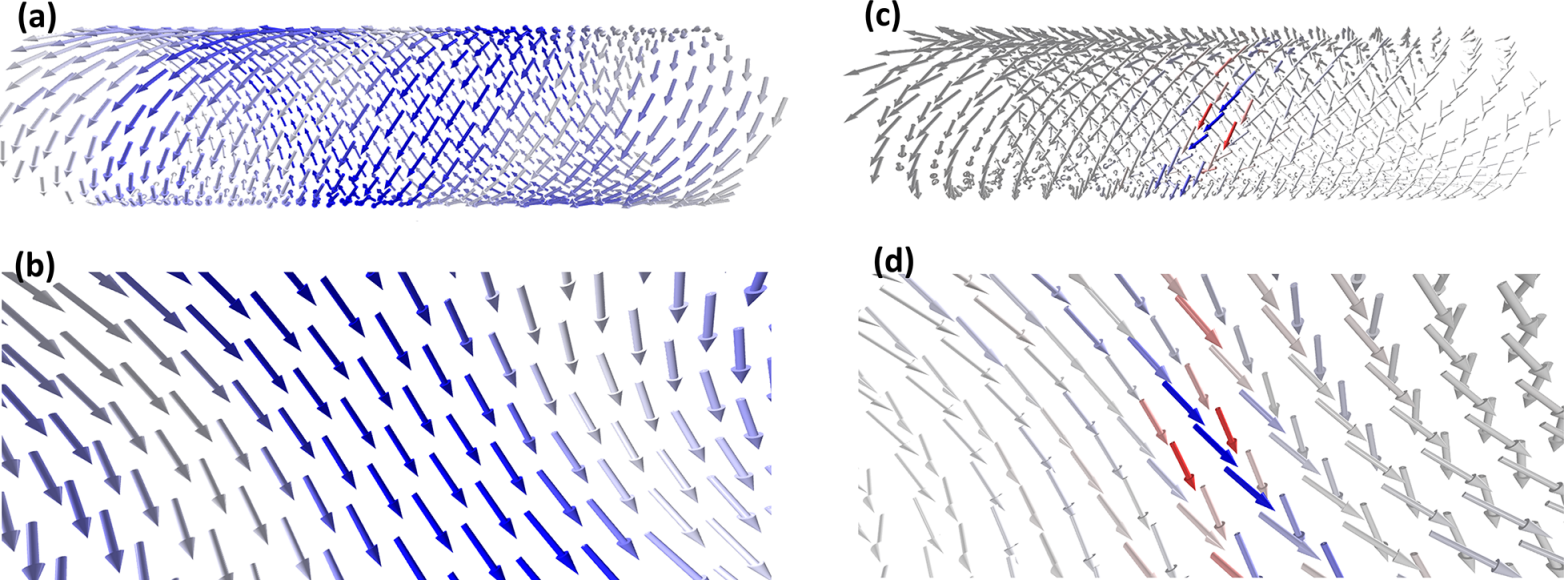
Illustration of the nature of the excitons in the BL (a, b) and HB (c, d) models. Average over the excitons contributing to the simulated PL (in the range 590–613 nm where the PL peak is observed) is calculated using spectrally weighted density matrix given in eq S9 in the SI. Panels (b) and (d) are the same as (a) and (c) but zoomed in and viewed from inside the tubes. Colors illustrate the sign of the wave function relative to the selected site for which vector is illustrated. Blue shows the same sign, while red shows the opposite sign, and white/gray arrows indicate that a given site is unlikely to contribute to the same exciton wave function or that different excitons included, destructively interfere when averaged. The nonlinear color scaling scheme *S*_n_ = tanh (3ρ_nm_^μ^/ρ_mm_^μ^) is used to highlight the low-weight contributions, where *m* is the label of a molecule in the center of the tube.

The theoretical analysis suggests that the experimentally observed angular and LDr distributions arise from the localization of the excitons on the microscopic level due to site energy disorder. For homogeneous (i.e., no site energy disorder) DWNT models, the symmetry considerations dictate that the excitons will always have transition dipoles either perfectly aligned along the tube axis or perpendicular to it. However, site energy disorder leads to symmetry breaking and partial localization of the excitons, which allows the transition-dipole moments of individual excitons to deviate from the ideal symmetry directions.^57^Figure 7 presents a visualization of such excitons.^58^ Analysis of the exciton wave functions contributing to the PL for the two structures (see SI, Section S9 for details) shows that the delocalization of the wave functions is hardly different between the HB and BL structures. The inverse participation ratio (eq S8) is essentially 225 in both cases for the exciton states in the range of the PL signal.

However, the structure of the excitons is very different due to the differences in molecular packing resulting in different signs of the couplings.^59^ In the HB structure, the largest coupling between chromophores is positive, but the combined effect of many smaller negative couplings still produces an overall redshift in the spectrum, similar to that observed in linear J-aggregates. In fact, the combination of positive and negative couplings for two-dimensional structures is characteristic of what is known as an RH-aggregate.^59^ The relevant excitons have both positive and negative wave function coefficients, which leads to destructive interference resulting in overall smaller transition dipole vectors that are less well aligned with the tube axis (exciton transition-dipole scatter plots are given in Figure S17).

In contrast, for the BL structure the dominant couplings are negative, which also results in an overall redshift of the spectrum (known as a RJ-aggregate behavior for two-dimensional structures^59^). This coupling pattern is similar to that recently reported for chlorosomes, light-harvesting complexes from green sulfur bacteria with the structure reminiscent of DWNTs.^60^ For the relevant exciton states in the BL structure, the wave function coefficients on all chromophores have the same sign, leading to superradiant states. The overall exciton transition dipole-moment, given by a weighted sum of all the transition dipoles contributing to the exciton wave function, is aligned very well with the tube axis.

## Conclusions

In this paper, we resolved a long-standing debate on the structural model, BL or HB, for C8S3-based supramolecular DWNTs. The combination of molecular-level exciton models, quantum-classical spectral simulations and optical microscopy on single objects, allowed us to identify LDr as a suitable observable to benchmark different structural models, in contrast to recent work.^51^

To test the theoretical predictions, we introduced a novel microscopy approach for measuring LDr by polarization-resolved PL measurements on individual DWNTs. Additionally, we developed a fabrication technique for strongly diluted DWNT samples, consisting of essentially a monolayer of immobilized DWNTs, which significantly minimizes out-of-focus, unpolarized background signals. This altogether allowed us to directly demonstrate that the transition dipole moments of the emitting excitons are aligned within a narrow angular range along the long axis of the DWNTs. Furthermore, we measured an unprecedentedly high median LDr value of 0.93, providing clear support for the BL structural model.

We found that the differences in LDr between the two models stem from the differing stacking arrangements, leading to distinct patterns in intermolecular electronic couplings. These, in turn, give rise to a very different nature of the exciton states contributing to PL. In the BL model, the largest couplings are negative resulting in exciton states, where all wave function coefficients are in phase. This leads to large transition dipole moments that are aligned closely along the tube axis and that give rise to PL nearly exclusively polarized along this axis. For the HB model the largest couplings are positive, while smaller couplings are negative, and the overall sum of the couplings is negative. The resulting exciton states have coefficients with alternating sign leading to transition-dipoles and emission, aligned less well with the tube axis.

Based on the results of this study, an intriguing question emerges: Can the BL model adequately describe the linear dichroism of higher-energy excitonic transitions? This question is particularly compelling because, as we have argued above, PL arises from the lowest-energy excitonic states, whose exciton wave functions retain high symmetry even in the presence of energetic and coupling disorder. In contrast, absorption involves transitions from the ground state to all optically allowed excitonic states, each possessing distinct symmetries. While directly measuring the absorption spectrum of a single DWNT remains beyond current capabilities, obtaining the polarization-resolved excitation spectrum using PL detection^61^ seems to be a feasible alternative. Moreover, exploring optical absorption circular dichroism in individual DWNTs presents an exciting next step, though it poses significant experimental and theoretical challenges. For instance, Bertocchi et al.^39^ reported an absorption circular dichroism Δ*A* = *A*_L_ – *A*_R_ (*A*_L,R_ is the absorbance for left- and right-handed circularly polarized light, respectively) of the order of 10^–4^ for thin films of C8O3-based aggregates. In the case of single DWNTs, we anticipate significantly smaller Δ*A* values, which remains beyond our current experimental capabilities. Furthermore, in both the BL and HB models, the exciton wave functions wind helically around the nanotube, likely leading to very similar circular dichroism spectra. Detailed and precise theoretical simulations are therefore needed to determine the extent to which distinct circular dichroism spectra may arise for single nanotubes with HB and BL packings.

Our findings offer valuable insights into the mechanisms governing excitonic transitions, particularly in nature-inspired synthetic light-harvesting complexes. These results are especially relevant for refining theoretical and computational models of excitonic processes in biological and bioinspired light-harvesting systems, such as photosynthetic complexes. Such systems depend on efficient energy transfer to achieve their remarkable light-harvesting efficiencies, making a molecular-level understanding of these processes essential for the development of biomimetic designs. Additionally, our study underscores the potential of stacking engineering as a powerful tool for fine-tuning exciton properties in supramolecular assemblies. By manipulating parameters such as supramolecular organization, it becomes possible to optimize PL polarization properties. This opens up new opportunities for the design of next-generation materials with tailored optical characteristics for practical applications, inspired by the efficient energy transfer mechanisms found in nature.

## Materials and Methods

### Calculations of the Spectroscopic Properties

Calculations of the spectroscopic properties of the two competing C8S3 inner wall structures were carried out using a quantum-classical approach. The aim is to compare the HB^30^ and BL^16,34^ structure models to the fullest extent using identical parameters for the spectral modeling. The obvious exception is that the molecular positions and transition dipoles were taken from each specific structural model and a scaling of the couplings in the BL model was applied to match the spectral position. The latter scaling factor is justified by the fact that the parameters for the extended transition-coupling model were part of the fitting procedure for the HB model. We thus allow the same freedom in our model for the BL structure of the coupling as was applied during the construction of the original HB model.^30^ The model for the dynamic excitation energy disorder of the individual C8S3 molecules is based on the previous multiscale modeling approach^32^ used for full DWNTs. The length of both modeled tubes is about 25 nm. A summary of the structural models taken from ref (30) and refs (16) and (34) for the HB and BL models, respectively, can be found in Section S1 of the SI. The two structures used are shown in Figure 1b and in more detail in Figure S1. The procedure to construct the exciton Hamiltonian trajectory that describes the interaction between the molecules and the fluctuating molecular disorder is provided in Section S2 of the SI. PL spectra were calculated using response function theory^62,63^ by averaging over spectra calculated along the exciton Hamiltonian trajectory. The details are given in Section S2 of the SI.

### DWNT Aqueous Stock

DWNT aqueous stock was prepared from 3,3′-bis(2-sulfopropyl)-5,5′,6,6′-tetrachloro-1,1′-dioctylbenzimidacarbocyanine (C8S3), obtained from FEW Chemicals GmbH under the name S0440, in the form of a powder. The powder was further purified through high-performance liquid chromatography (HPLC, performed at Freie Universität Berlin, Institute of Chemistry and Biochemistry). DWNTs were prepared via an alcoholic route: C8S3 was dissolved in MeOH at a concentration of 2.1 g/L, and 1 mL of milli-Q H_2_O was added to 260 μL of the MeOH stock solution.^28^ The mixture was gently stirred and left in a dark place at 20 °C with 40% relative humidity for ∼12 h to facilitate DWNT formation.

### UV–Vis Absorption

UV–vis absorption spectra were measured in solution in a 50 μm thick cuvette in a UV–vis spectrometer (UV-2600i, Shimadzu).

### PL Emission

PL emission spectra were measured in solution in a 1 cm thick cuvette in the LS50B spectrofluorometer (PerkinElmer).

### Single-DWNT Microscopy

For excitation, a continuous-wave semiconductor laser (Coherent) operating at a wavelength of 561 nm was used (SI, Figure S2). The laser’s linear polarization was converted to circular polarization (SI, Figure S3) to ensure uniformity of excitation. Additionally, the wavelength of 561 nm is close to the known zero point of LD,^27^ ensuring uniform excitation of DWNTs regardless of their orientation. Ultrafast population relaxation transfers the initially excited high-energy excitons to the main low-energy transitions within a 100 fs time scale.^64,65^ The excitation light was focused onto the sample using an air objective (20×, numerical aperture NA = 0.2), creating a focal spot with a diameter of ∼10 μm and an excitation power of ∼1 μW. Immersion oil (*n* = 1.518, Olympus Type F) was applied on top of the sample to eliminate depolarized interface-reflected PL, which could negatively affect the PL polarization measurements.

The PL was collected in transmission mode using an oil-immersion objective (PlanApoN, NA = 1.42, Olympus). Immersion oil was chosen to be Olympus Type F, *n* = 1.518. The PL was spectrally filtered by a 590 nm long-pass filter (590 LP ET, AHF Analysentechnik) to suppress laser light as well as the contribution of the PL from the outer tube thereby ensuring that the detected PL originated predominantly from the inner tube (SI, Figure S4). Next, the PL passed through a half-wave plate (400–800 nm Achromat, Thorlabs) mounted on a computer-controlled rotation stage. A Wollaston prism (1°20′ separation angle, Thorlabs) was placed in front of a CMOS camera (Chameleon3, Teledyne FLIR) to spatially separate the two orthogonally polarized images. The precise orientation of the half-wave plate was verified with a vertically polarized He–Ne laser with an ∼1° accuracy. The detection path was carefully checked to be fully isotropic, with no optical elements introducing undesired anisotropy (SI, Figure S10). The optical resolution of the microscope was determined to be ∼160 nm (standard deviation of a Gaussian fit) based on imaging of a DWNT cross-section (SI, Figure S5).

The schematic of the microscopy setup, data acquisition and processing protocols are provided in the SI, Sections S3–S5.

### Thin Samples

Thin samples were prepared by suspending the DWNTs in an aqueous sugar solution, following refs (25) and (66) with some modifications. Sucrose and trehalose (Sigma-Aldrich) were dissolved in water at concentrations of 1 and 0.5 g/mL, respectively. Both solutions were mixed in a 1:1 volume ratio at room temperature to form the sugar stock solution. Then, 1–4 μL of DWNT was added to 400 μL of the sugar stock, and the mixture was stirred thoroughly. A 10 μL droplet of the mixture was deposited onto a glass microscope coverslip and subsequently dried by gradually increasing the nitrogen flow. First, a weak nitrogen flow was used to push the body of the droplet aside, leaving a thin residue trace near the center of the glass slide. Next, the nitrogen flow was increased 10-fold to remove the bulk of the droplet from the slide and simultaneously dry the droplet trace, forming a solid film containing DWNTs incorporated into the sugar matrix. Such argon-flow drying technique enabled the formation of DWNT films as thin as 1.4 μm (SI, Section S6). Such thickness allows the DWNTs to achieve an almost coplanar orientation, ensuring that all DWNTs within the field of view were in focus. The sample concentration of DWNTs was kept low enough to maintain spatial separation of the DWNTs from each other in all directions, allowing for single DWNT imaging.

DWNTs stabilized in the sugar matrix are known^25,67^ to exhibit spectra similar to those observed in aqueous solution (Figure 1c). All operations involving DWNT sample preparation were performed in the dark.

## Supporting Information Available

Structural models; Spectral calculations; Details of the microscopy setup; Data acquisition; Data processing; Sample thickness measurements; Additional Theoretical Analysis; PL intensity profiles analysis; Delocalization size. Additional references cited in the SI (PDF)
