# Supplementary material for: Linear Dichroism Microscopy Resolves Competing Structural Models of a Synthetic Light-Harvesting Complex
Source: J Am Chem Soc. 2025 Feb 4;147(7):6171–80. doi: 10.1021/jacs.4c17708 (PMC11848924; doi:10.1021/jacs.4c17708)
Supplement: Supplementary file 1 — ja4c17708_si_001.pdf [file ja4c17708_si_001.pdf]

Supporting Information

Linear Dichroism Microscopy Resolves Competing Structural Models of a Synthetic Light-Harvesting Complex

Alexey V. Kuevda [a], Mónica K. Espinoza Cangahuala [a], Richard Hildner [a], Thomas L.C. Jansen [a]*, and Maxim S. Pshenichnikov [a]*

[a] Zernike Institute for Advanced Materials, University of Groningen, Nijenborgh 3, 9747 AG Groningen, The Netherlands

* Email: t.l.c.jansen@rug.nl

* Email: m.s.pchenitchnikov@rug.nl

Table of Contents SI

Supporting Information 1

Section S1. Structural models 2

Section S2. Spectral calculations 3

Section S3. Details of the microscopy setup 5

Section S4. Data acquisition 7

Section S5. Data processing 8

Section S6. Sample thickness measurements 12

Section S7. Additional Theoretical Analysis 13

Section S8. PL intensity profiles analysis 16

Section S9. Delocalization size 17

References 20

Section S1. Structural models

The C8S3 inner wall (IW) nanotube was modelled using: 1) the Herringbone (HB)[1] model, and 2) the bricklayer (BL) model[2,3]. In general, the structural models of individual tubular aggregates can be constructed by wrapping two-dimensional sheets of molecules around a cylindrical structure[1,4]. In 2012 Eisele *et al.* [1] reported a HB model that correctly reproduced the experimental absorption spectrum (energy positions and polarizations of the four transition bands) of the isolated C8S3 IW DWNT. We used this HB structure model to obtain the structure of the IW that is 25.0 nm long with a radius of 3.6 nm. The parameters used are tabulated in Table S1.

Table S1. Structural parameters used to construct the inner wall using the HB structure model as described in Eisele *et al.* [1]. The parameters are radius (R), (nanotube) length (L), Number of monomers (N), Number of rings (N_1), height (length) parameter (h), and chiral vector parameters t_1 and t_2. The equations describing the positions and transition dipole moment vectors are given in Eq. S1 and S2 of that paper.

Parameters*	Inner Wall
t_1	18
t_2	12
R (nm)	3.551
N_1	84
N	1008
h (nm)	0.296
L (nm)	24.8
β (°)	56.7

For the BL model we used the high-resolution cryo-EM imaging structure[2] made up of an asymmetric unit (ASU) with C_5-rotational symmetry containing six molecules per ASU. The coordinates file for the inner wall structure can be found in the supplementary information of Ref.[2] We base our BL model on this IW structure by extracting the central atom coordinates of each molecule in the IW structure (780 total) and using those coordinates to center the position of the dipoles in the BL model of the IW. The resulting IW cylindrical structure is 25.8 nm long

with a diameter of 3.8 nm. A schematic representation of both structural models is shown in Fig. S1.

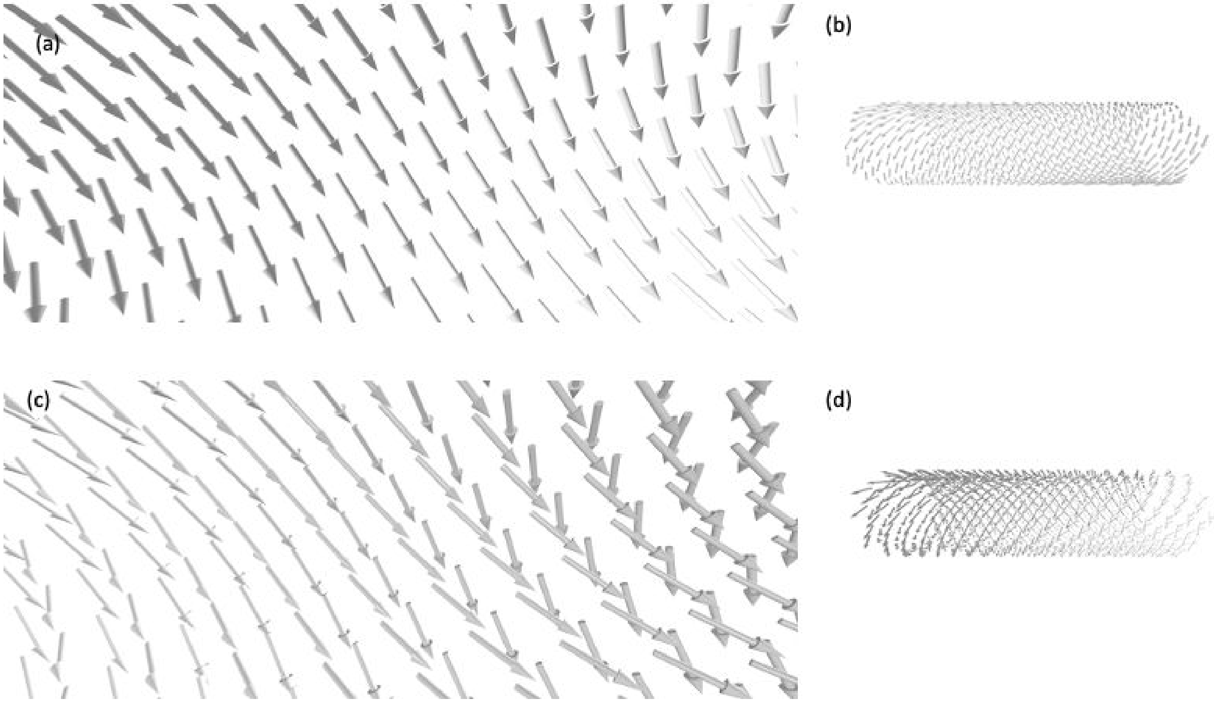

Figure S1: Structure of the BL model from Ref.[2] (a and b) and the HB model from Ref.[1] (c and d). The transition dipoles are illustrated with arrows pointing from the middle of each C8S3 molecule. Panels (a) and (c) show close-up views of the packing as seen from inside the tubes, while (b) and (d) show the full simulated tubes. The BL structure is 25.8 nm long, while the HB structure is 25.0 nm long. The radii are 3.8 nm and 3.6 nm, respectively. The BL structure contains 780 molecules, while the HB structure contains 1008 molecules.

Section S2. Spectral calculations

We model the absorption and luminescence spectra using the Frenkel exciton Hamiltonian, which has previously been used to describe the electronically excited states in C8S3 and other molecular aggregates[5]. When setting $\hbar$ equal to one, the Frenkel exciton Hamiltonian is:

$$H = \sum_{n} \omega_n b_n^\dagger b_n + \sum_{n,m\neq n} J_{nm} b_n^\dagger b_m \tag{S1}$$

Here, the molecular excitation energy for each chromophore, ω_n, is given by the average gas phase energy 18868 cm^{-1}. Realistic dynamic site energy disorder was generated using two

uncorrelated overdamped Brownian oscillators[6] for each C8S3 molecule, with 83 and 208 cm^{-1} as the standard deviation of the fluctuations and 1000 fs and 46 fs as the timescales for the two components, respectively[7]. These parameters reproduce the dynamics determined in the previous multiscale modelling study[8]. The couplings between chromophores were obtained with the already well-established extended dipole coupling model[3,9], where the molecular excitations are treated with transition-dipoles, $\vec{\mu} = q\vec{l}$ using transition-charges of magnitude $\pm q$ displaced from each other by the distance l (0.7 nm) [3]:

$$J_{nm} = A\frac{\mu^2}{l^2}\left[\frac{1}{r_{nm}^{++}} - \frac{1}{r_{nm}^{+-}} - \frac{1}{r_{nm}^{-+}} + \frac{1}{r_{nm}^{--}}\right] \tag{S2}$$

where A is a conversion constant (5.04 $cm^{-1}nm^3Debye^{-1}$), μ is the transition-dipole moment (11.4 Debye). The resulting transition-charges (0.34e) are displaced from the central atom position along the transition-dipole vector, which is largely along the polymethine bridge[3]. $r_{nm}^{\pm\pm}$ are the distances between the positive and negative transition-charges at molecule n and m. Additionally, for the BL model the coupling was scaled by a factor 0.75, which may be justified by the fact that the original parameters of the extended transition-dipole coupling model were fitted for the HB model. This is also in line with a molecular transition dipole moment around 9.6 Debye as recently used for the similar C8O3 dye[10]. This approach was used to construct a 6 ps long Hamiltonian trajectory with snapshots stored every 3 fs.

The overall PL was determined as the average of the signal in each cartesian direction α using the response function expression:

$$I_\alpha(\omega) = Re\int_0^\tau \left\langle \mu_\alpha(t)U(t,0)\mu_\alpha(0)\exp\left(-\frac{H(0)}{k_BT}\right)/Z\right\rangle \exp(-(i\omega+\Gamma)t)\,dt \tag{S3}$$

Here, μ_α is the molecular transition-dipole, $U(t,0)$ is the time-evolution operator, k_BT is the thermal energy needed in the Boltzmann weight, and Z is the partition function. Γ is an artificial decay used for smoothening with $\Gamma = 20$ $ps^{-1}$and 5 ps^{-1} for the BL and HB models, respectively. $\tau = 192$ fs is the longest time for which the response function was calculated. The calculations were performed using the NISE approach[11,12], where the time-evolution operator is determined by dividing time into short (0.3 fs) intervals[13] and using that the Hamiltonian is

changing slowly enough that it can be assumed fixed within the short interval. The reduced linear dichroism of the PL was determined using Eq. 2 of the main text.

Section S3. Details of the microscopy setup

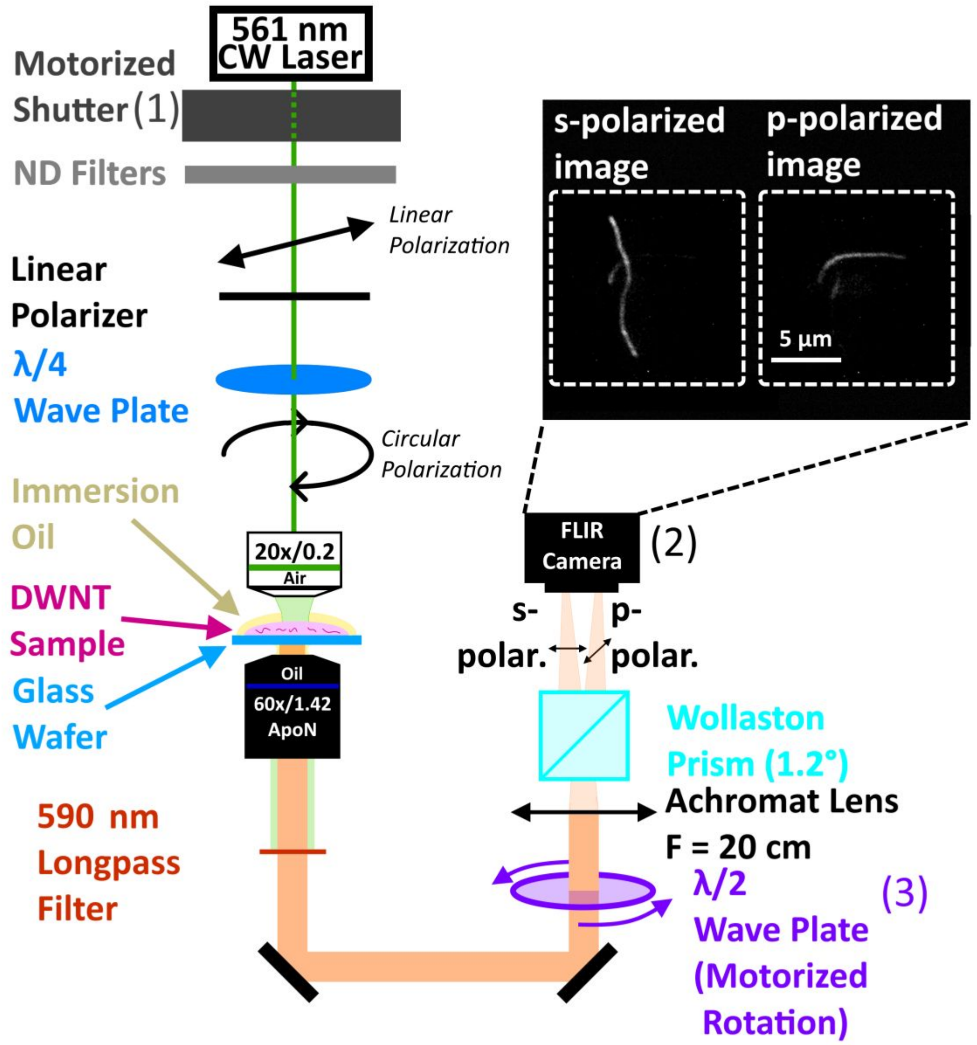

Figure S2. Schematics of the microscopy setup for polarization-resolved PL imaging. The inset shows *s*- and *p*-polarized images of two representative DWNTs. Note that we used a 1°20' separation angle Wollaston prism (Thorlabs) as the higher separation angle (>5°) Wollaston prism leads to a potentially detrimental effect of shifting the focal planes of each polarization differently[14]. The following elements of the setup were fully computer-controlled: the laser shutter (1), the CMOS sensor (2), and the half-waveplate rotator mount (3). This automation enables the collection of extensive statistics on DWNTs while minimizing their exposure to potentially photo-damaging laser excitation.

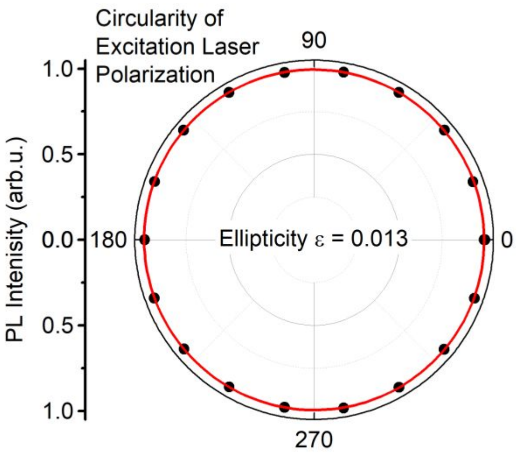

Figure S3. Characterization of the polarization of the excitation source. A linearly polarized semiconductor laser with a wavelength of 561 nm was used with its linear polarization reenforced by a thin-film polarizer. A zero-order quarter-wave plate (WPQ05M-561, Thorlabs) was then inserted into the laser beam to achieve circular polarization. The circularity of the beam polarization was verified by rotating a half-wave plate in front of the Wollaston prism while imaging a 25 μm pinhole. The laser intensity was recorded as a polar plot, showing fitted (red line) ellipticity (i.e., deviations from a perfect circle) of $\varepsilon = 0.013$. ~~.~~

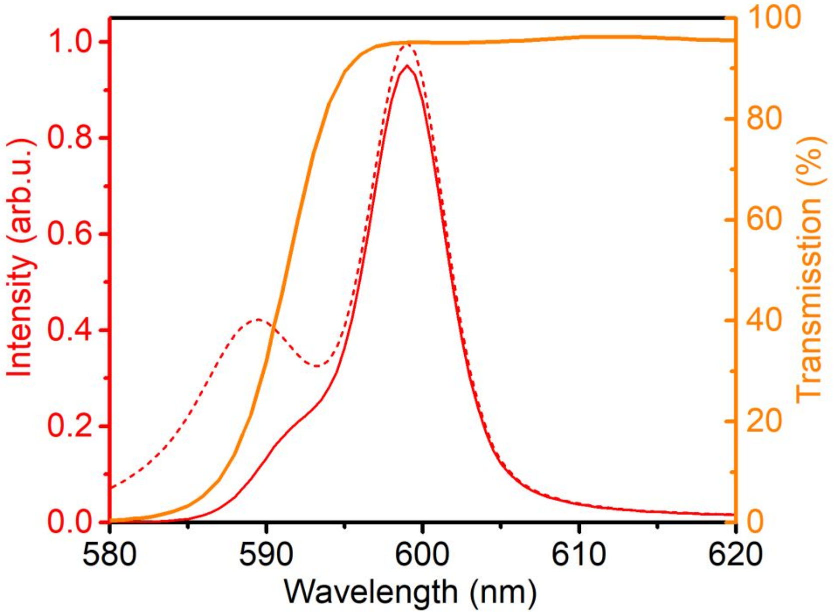

Figure S4. Filter transmission spectrum (orange), used to block the laser excitation light and PL originating from the outer wall of DWNT at 588 nm. The dashed and solid red curves show the DWNT PL spectrum before and after the long-pass filter, respectively. The ~~detected~~ PL spectrum indicates that the PL primarily originates from the inner wall of the DWNT.

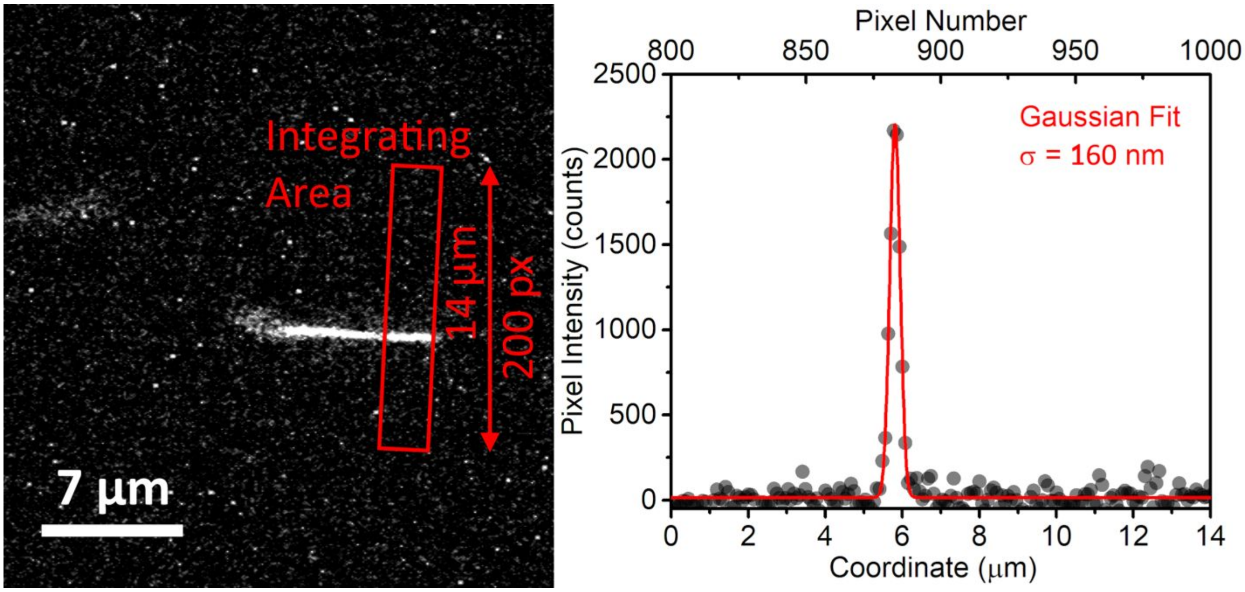

Figure S5. Optical resolution of the microscope determined by Gaussian fitting of the DWNT cross-section.

Section S4. Data acquisition

First, a sample area containing 1-5 spatially separated coplanar DWNTs was manually selected, and the focus adjusted (Fig. S2). Then, the measurement script was initiated (Fig. S6). The software opened the laser shutter to illuminate the sample, and the CMOS sensor simultaneously began accumulating photons for a defined exposure time (typically 10-500 ms). Each frame captured by the CMOS sensor thus comprises two parts: the left part corresponded to an *s*-polarized PL image of DWNTs, while the right part corresponded to a *p*-polarized PL image (in the laboratory system of coordinates). This approach effectively doubles the amount of information obtained during the same exposure time. The sum of these two images forms the isotropic image. Then the shutter was closed, the half-wave plate was rotated by the specified angle step $\Delta\alpha$, which caused the polarized PL to rotate by $2\Delta\alpha$, and the cycle was repeated until the complete PL polarization orientation diagram is recorded. Typically, each measurement involved a complete rotation of the wave plate, resulting in two full rotations of the PL signal (0° to 720° with a $2\Delta\alpha = 20°$ step).

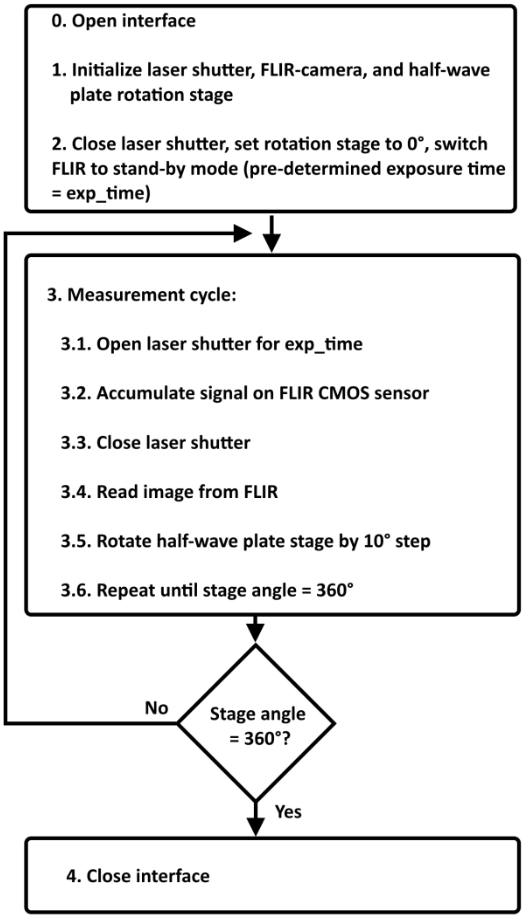

Figure S6. The algorithm of image acquisition. Script-controlled parts are the laser shutter, the CMOS-camera, and the half-wave plate rotation stage (1, 2, and 3, respectively in Fig. S2).

Section S5. Data processing

The set of images was processed using ImageJ software[15] (LOCI, University of Wisconsin). The process began by applying a mask to the straight segments of the DWNT image and extracting the integral signals from the selected area for each half-wave plate orientation. The same area mask was then applied to a dark, DWNT-free region to subtract background signal. This procedure was applied to both *s*- and *p*-polarized images, producing two sine-like, phase-shifted signals (Fig. S7a). Next, each integrated signal was normalized to the sum of the two to account for any photobleaching-induced degradation of the PL signal (Fig. S7b). For the polarized PL, this procedure resulted in two sinusoidal traces, phase-shifted by $\pi/2$. The minimum and maximum values needed to calculate LDr were determined by fitting the obtained traces (Fig. S7c) using the following formulae:

$$
\begin{aligned}
I_{\parallel}(\alpha) &= \left(I_{\parallel}^{Max} - I_{\parallel}^{Min}\right)\cos^2[2(\alpha - \alpha_c)] + I_{\parallel}^{Min} \\
I_{\perp}(\alpha) &= \left(I_{\perp}^{Max} - I_{\perp}^{Min}\right)\sin^2[2(\alpha - \alpha_c)] + I_{\perp}^{Min},
\end{aligned}
\tag{S4}
$$

where $I(\alpha)$ represents the angle-dependent PL intensity, α is the half-wave plate orientation angle, $I_{\parallel,\perp}^{Max}$ and $I_{\parallel,\perp}^{Min}$ are the maximal and minimal PL intensities, respectively, in the parallel and orthogonal channels, and α_c is the angle at which the PL reaches its maximum value in the parallel channel, and its minimum in the orthogonal channel. The α_c value was used to directly determine the PL polarization orientation from the PL images with an accuracy of ~1° (Fig. S8).

The experimental LDr values were calculated according to Eq. 2 in the main text as

$$
\mathrm{LDr} = \frac{I_{\parallel} - I_{\perp}}{I_{\parallel} + I_{\perp}} \tag{S5}
$$

The minimum and maximum values for each polarization in Eq. S4 are transformed into the LDr values in Eq. S5 as

$$
\mathrm{LDr} = \frac{I^{Max} - I^{Min}}{I^{Max} + I^{Min}} \tag{S6}
$$

The LDr values obtained from two channels were averaged to obtain the final LDr value.

To align the images captured by the camera with the actual DWNT sample positioned in the sample plane, the camera image was rotated π/2 clockwise and flipped along the vertical axis following the image formation in the detection path of the microscope. The orientation of the DWNTs was measured using image-editing software (Adobe Photoshop). The same DWNT segments previously selected for LDr analysis (Fig. 4 of the main text) were fitted with straight lines. These lines were drawn to connect the midpoints of the cross-sections at the start and end of each DWNT segment. The angle of the drawn lines was measured counterclockwise from the horizontal line.

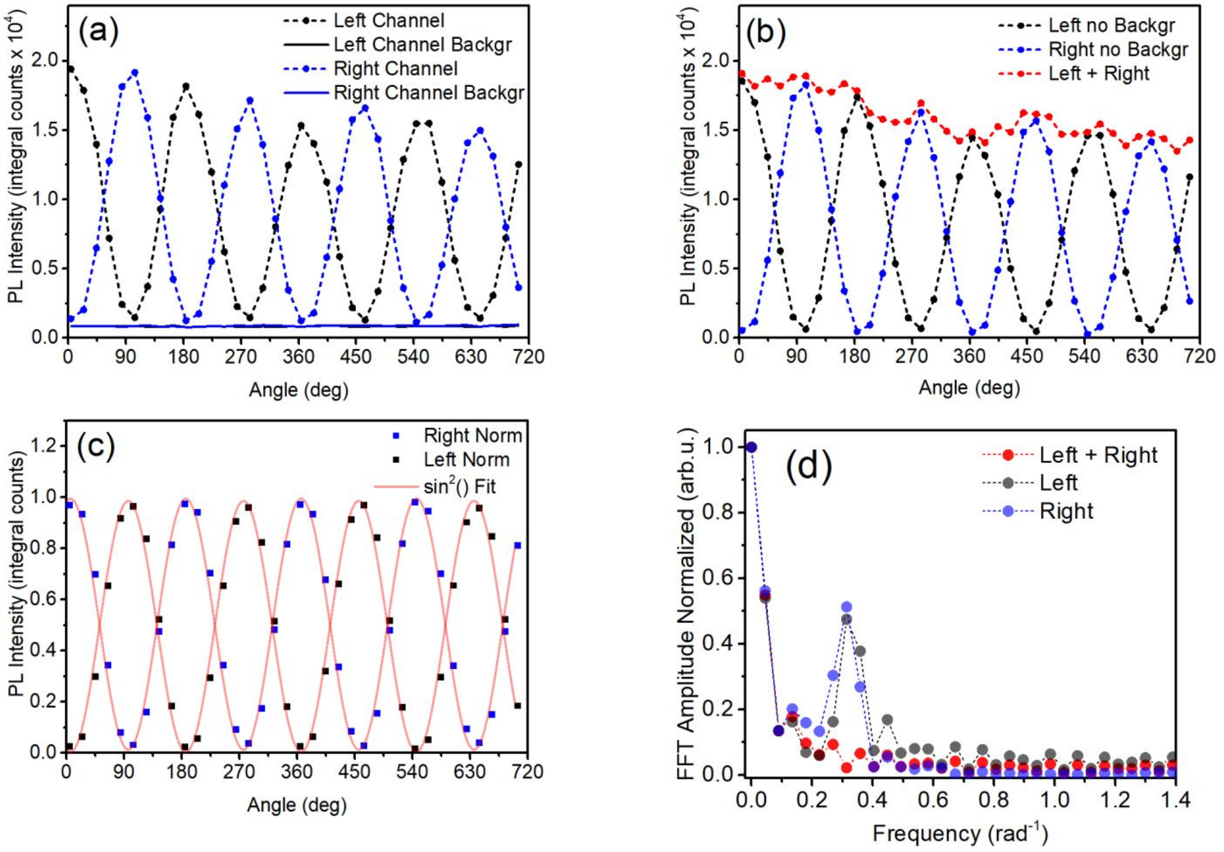

Figure S7. The data processing procedure applied to each individual DWNT included in the LDr dataset. As an example, the data for DWNT1 in Fig. 4 of the main text are shown. (a) Raw PL intensity values from DWNT1, extracted using an identical image mask in both the left and right channels (black and blue points connected by a dashed line). The same mask was applied to adjacent areas in each channel, which contained no DWNTs or other PL emitters, providing the background signal (solid curves). (b) The same data with the background signal subtracted. Red dots represent the sum of the signals from the right and left channels, i.e. the unpolarized PL intensity. This summed PL signal decreases over time due to sample photobleaching caused by the excitation laser. (c) Polarization-resolved signals (dots) normalized by the sum signal to completely remove photobleaching effects. The solid red lines represent the sine-squared signals, from which the polarization orientation angle and LDr are directly extracted. (d) The Fourier spectra of the sum signal (red), right (blue) and left (black) channels from the panel (b). The π-modulation peak, clearly visible in both spectra of the left and right channels, is completely eliminated in the summed signal.

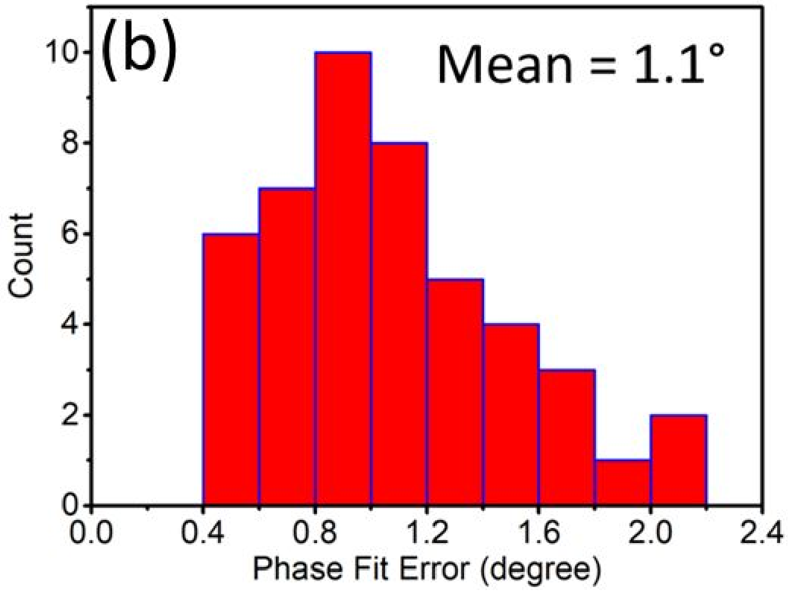

Figure S8. The analysis of phase shift fit uncertainty (as obtained from the fits) in the PL polarization curves for all analyzed DWNTs. The results show that the positions of the PL polarization maxima/minima are determined with an accuracy of 1°.

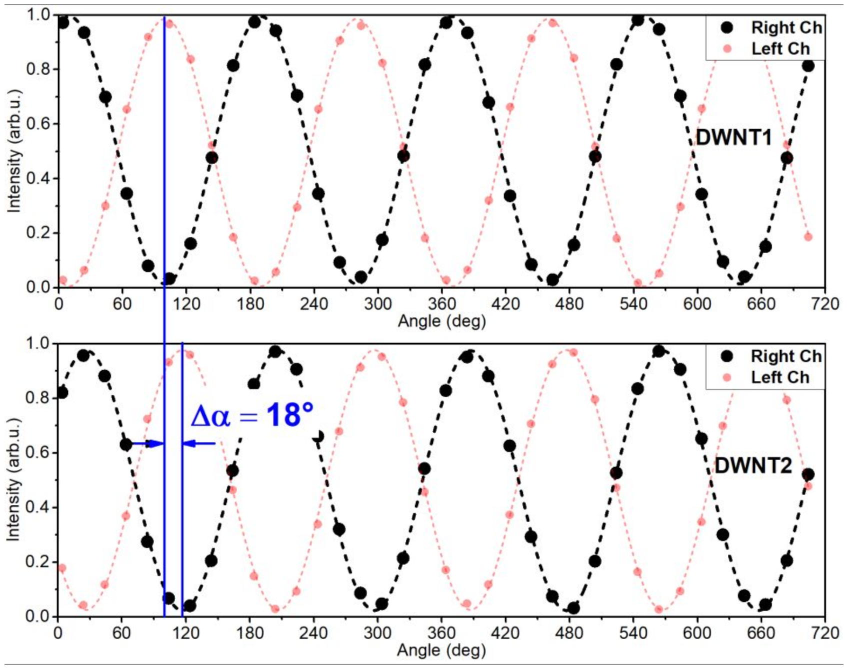

Figure S9. PL intensities of DWNTs shown in Fig. 4 of main text processed as described above, as a function of PL polarization rotation angle for DWNT1 (top panel, red in Fig. 4) and DWNT2 (bottom panel, green in Fig. 4). Red and black dots represent experimental data points, while the lines correspond to the respective cosine squared fits. The extracted value of the angle difference of 18° matches well the angle of 17° determined from the geometrical orientations of the DWNTs.

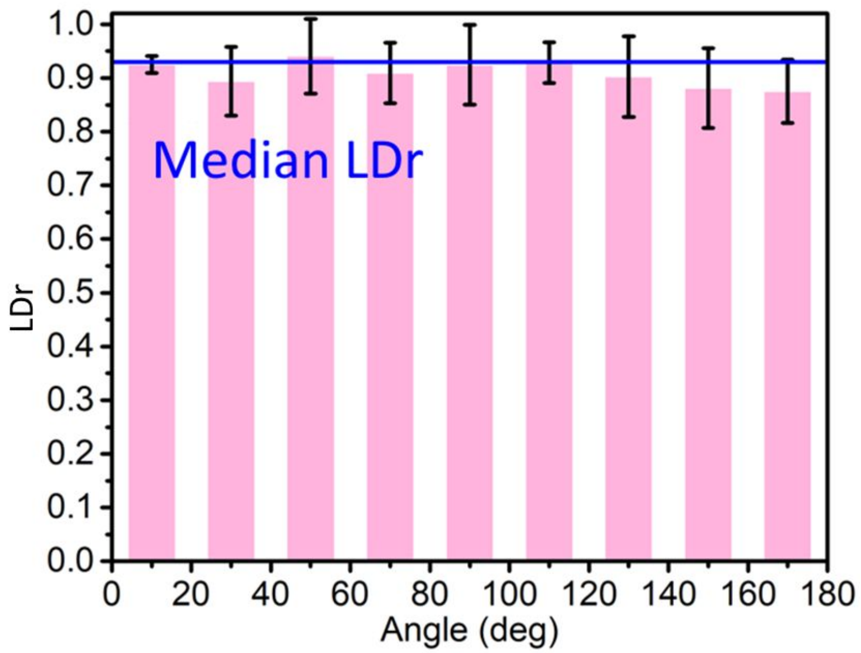

Figure S10. Independence of LDr with respect to the DWNT orientations. The histogram displays the median LDr values averaged over 20° bins. The solid line represents the median value obtained from the histogram (Fig. 6) in the main text. The LDr values show no dependence on DWNT orientation, due to the uniform excitation provided by circularly polarized light and the polarization isotropy of the detection path.

Section S6. Sample thickness measurements

Film thickness was measured using a DektakXT Bruker profilometer equipped with a 12.5 μm stylus radius tip, and a stylus force set at 1 mg. Thin-film samples were prepared as described in the Materials and Methods section under the "Thin Films" subsection. To measure thickness, thin traces were scratched through the film to the glass substrate using a scalpel blade at five randomly selected positions arranged in a grid. This procedure was repeated on four different samples. Fig. S11 presents two representative scratches, highlighted by red dashed lines in the photograph, alongside a corresponding profile scan.

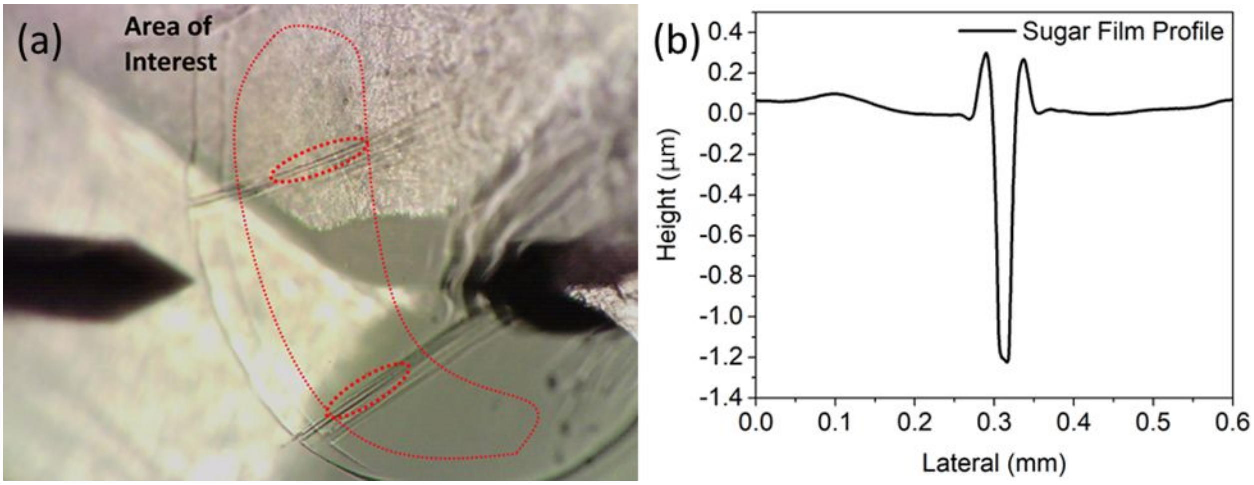

Figure S11. Measurement of the sample thickness. (a) Photograph of the profilometer setup used to extract profiles of incision scratches made on ultrathin sugar matrix samples. The close-up image shows the ultrathin sugar film with scratches across its surface. The red dashed line highlights the area containing two representative scratches used for profile measurements. (b) A representative profile extracted from the measurement, illustrating the thickness of the sample.

Table S1. Thickness measurements from five different areas of four thin-film sugar matrix samples. The median sample thickness is 1.4 ± 0.7 μm.

Thickness, μm			
sample 1	*2*	*3*	*4*
1.2	1.6	3.3	1.4
1.2	1.6	3.4	1.4
1.3	1.6	2.5	1.2
1.2	1.3	2.6	1.4
1.2	1.3	2.7	1.4

Section S7. Additional Theoretical Analysis

To validate the choice of the wavelength cut-off on the red side of the spectrum used in Fig. 3 of the main text the analysis was repeated with three different cut-off values. The resulting data are compared in Fig. S12. It is clearly seen that the choice of the cut-off does not matter for the interpretation.

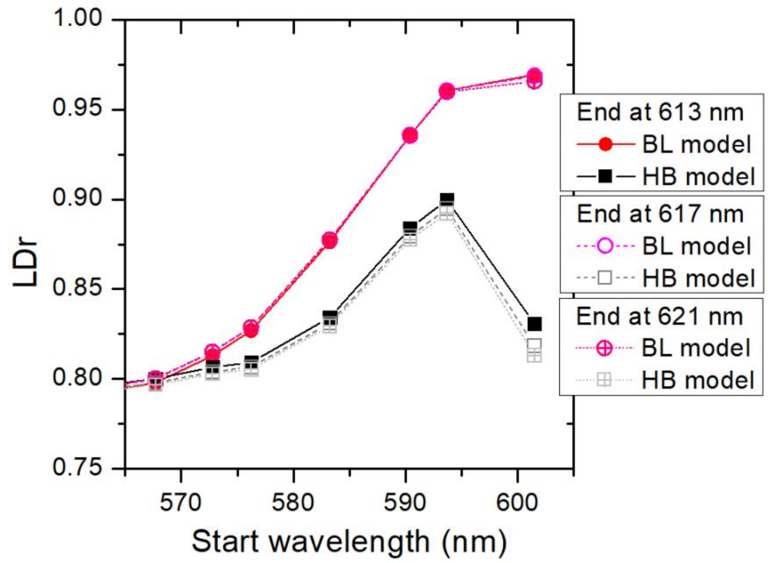

Figure S12. Calculated LDr values of simulated PL spectra for various spectral integration ranges. LDr averaged in the range from the wavelength shown in the graph to the wavelength shown in the legend. The wavelengths are shifted by 12 nm as in Fig. 2 of the main text.

The origin of the large difference in the LDr between the two model structures was further analysed by examining the contributions from individual exciton states along the trajectories. This was done by calculating the LDr for each exciton state *i* using its transition-dipole moment components $\mu_{x,i}$,$\mu_{y,i}$, and $\mu_{z,i}$:

$$LDr^i = \frac{\mu_{z,i}^2 - \frac{1}{2}\left(\mu_{x,i}^2 + \mu_{y,i}^2\right)}{\mu_{z,i}^2 + \frac{1}{2}\left(\mu_{x,i}^2 + \mu_{y,i}^2\right)} \quad \text{(S7)}$$

In Fig. S13 a histogram of these LDr values is shown where each exciton state in the range of 15800 to 16600 cm^{-1} (621-590 nm, including the 12 nm shift) is weighted with the square of its transition-dipole and the Boltzmann weight determined from the exciton energy of the state. A bit of caution should be made when comparing with the similar graph of the distribution of experimental LDr values (Fig. 6 in the main text), as the experimental values reflect an average over an ensemble of exciton states and not the distribution of individual exciton states. Furthermore, exciton states with transition dipoles close to perpendicular to the tube axis contribute to a reduction in the LDr calculated from the full spectra as obtained with Eq. S3. The presented distributions, however, still reveal that for the HB model the underlying excitons have

a much larger spread in LDr than the excitons in the BL models do. The experimentally observed spread in angles can, thus, be traced back to the exciton level.

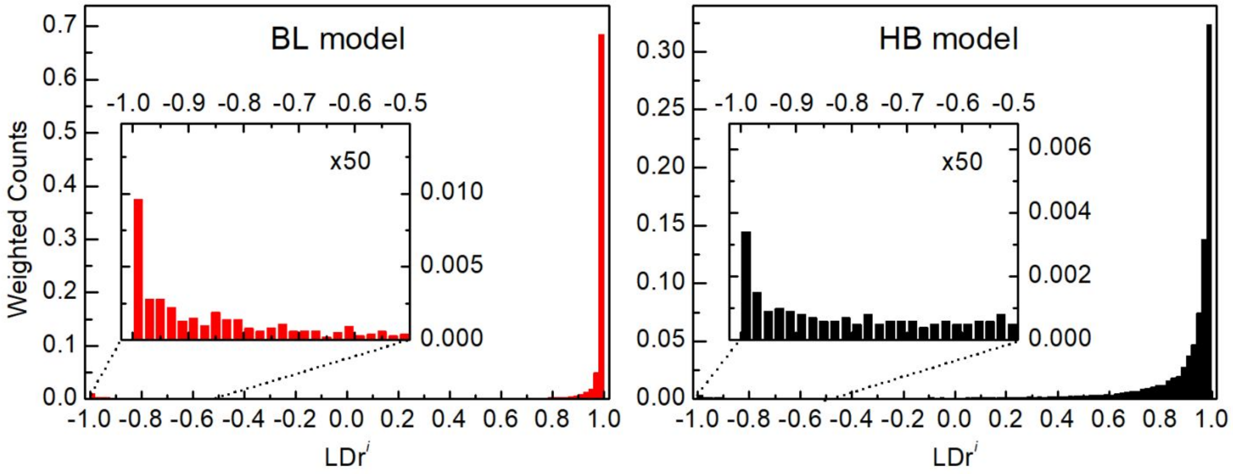

Figure S13. The distributions of the LDr^i contributions from individual exciton states for the two structural models. The bin size is 0.02. Insets highlight excitons with negative LDr^i values.

A more intuitive picture of the effect of site energy disorder on the angle distribution of the exciton state transition dipoles was made by mimicking the situation where the DWNT is lying flat on a surface. The angle between the transition dipole moment of each exciton state and the tube (z-axis) was determined in the xz- and yz-planes. The distributions weighted with the same factors as the LDr above are shown in Fig. S14. The width of the distribution for the BL structure is much narrower than that found for the HB structure. The width found for the BL structure is comparable with an angle found in the inset of Fig. 5b in the main text.

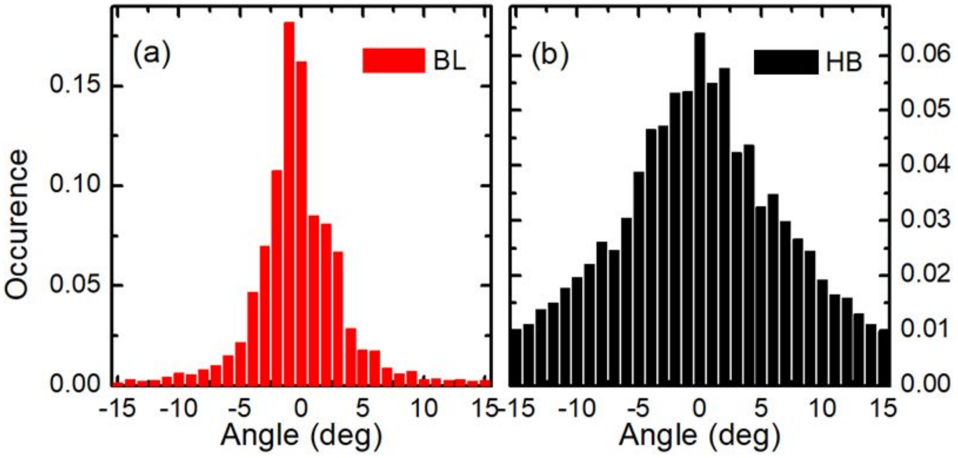

Figure S14. The distribution of the angles between transition-dipoles of individual exciton states with the tube axis, projected in a two-dimensional plane for BL (a) and HB (b) models. The exciton state contributions are weighed with their intensity contribution to the PL spectra. All histograms are normalized to the total of unity. Bin size is 1^0.

Section S8. PL intensity profiles analysis

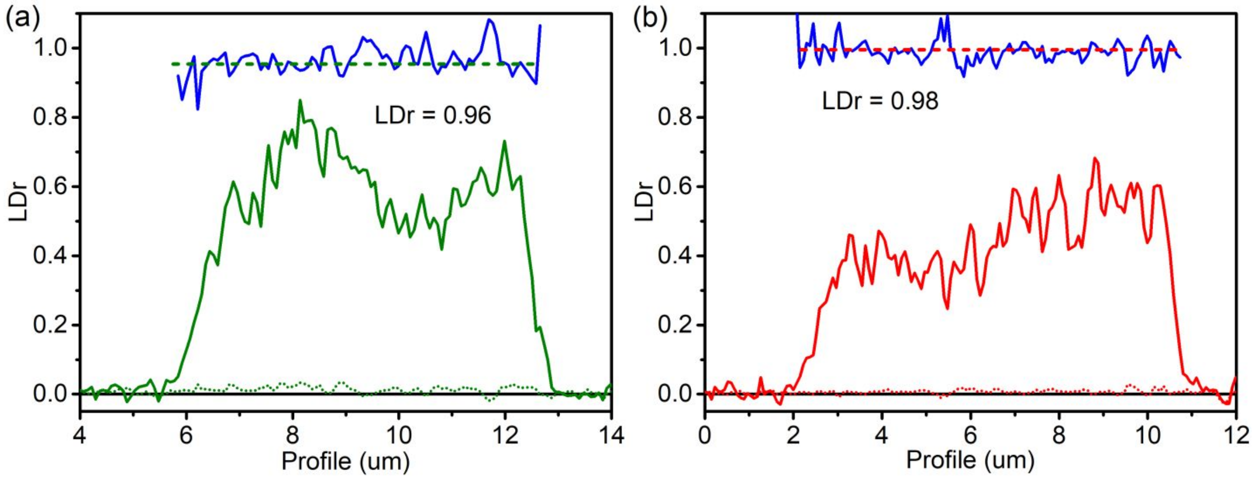

Figure S15. Normalized PL intensity profiles (red and green lines) and LDr values (blue lines) along the lengths of the two DWNTs shown in Figure 4 of the main text (the colors correspond to those in Fig. 4). Solid and dotted lines represent the profiles at the angles of the half-wave plate that provide maximum and minimum PL, respectively. Since the sampling angles of the half-wave plate are not perfectly aligned with the random DWNT orientations (because of the finite step size of the half-wave plate $\Delta\alpha = 10°$) , the PL profiles were extrapolated to their maximum and minimum values, as shown in Fig. S7c. The linear fits to the LDr values are represented by dashed lines, with the averaged LDr values indicated. Note that despite PL variations, the LDr values calculated along the DWNT length remain constant and consistent with the average values reported in Figure 5 of the main text.

Section S9. Delocalization size

The delocalization size was determined using the inverse participation ratio (IPR) [16]:

$$\mathrm{IPR} = \left\langle \sum_i \left(\sum_m |c_{im}|^4 \right)^{-1} \right\rangle \tag{S8}$$

Here, i labels the exciton states, m labels the molecular sites and c_{im} are the corresponding exciton wavefunction coefficients. The wedge brackets symbolize averaging over the trajectory with site disorder. The IPR averaged *over all exciton states* was found to be 239 and 322 for the BL and HB structures, respectively. For excitons with energies *in the range of 15800 to 16600 cm^{-1}* (621-590 nm, including the 12 nm shift), where the dominant PL peak is observed, the IPR is 224 and 226 for BL and HB, respectively. This suggests very comparable extents of the excitons. We note that the large delocalization lengths compared to the overall number of molecules indicate that in the current calculations we are close to the finite size limit. Unfortunately, no larger BL structure is available limiting us to the current sizes. Furthermore, the PL calculations are rather computationally costly for large systems. While one may want to take the IPR values with some caution, they still provide a reasonable way of comparing the two structures and for larger systems the delocalization lengths would only be larger. The finite size effect was previously shown to be most important in the middle of the band and examined in more detail for the HB structure[18].

For further analysis we calculated the spectrally weighted density matrix[17]

$$\rho_{nm}^{\mu} = \left\langle \sum_i |\mu_i|^2 c_{in}^* c_{im} \right\rangle \tag{S9}$$

This quantity reveals information about the nature of the exciton states contribution to the spectra. This full quantity is not easy to visualize in a meaningful way for a tubular structure and it is likely to contain a lot of redundant information due to the high symmetry between the different sites. Therefore, we instead show one representative vector centered at the middle of each of the tubes (Fig. 7 of the main text). The difference between these quantities is quite extreme. For the BL structure the exciton shape is winding around the tube in a helical way. All the coefficients of the illustrated vector are positive, revealing that the wavefunction coefficients

contributing to the strongly absorbing exciton states all have the same sign. This is a typical signature expected for a J-type aggregate. We indeed also observe that the couplings between different sites are predominantly negative (see Fig. S16).

For the HB structure the visualized vector is much more localized. There are just three clearly positive elements, but two or maybe three visibly negative ones. This reveals that the HB structure has an entirely different type of excitons. The two types can be roughly understood as so-called RJ- and RH-aggregates for the BL and HB structures, respectively, following the characterization of two-dimensional aggregates[18]. In essence, for the HB structure the largest couplings (Fig. S16) are positive resulting in an out of phase relationship in the wavefunction coefficients in the direction perpendicular to the transition-dipole directions. In the direction along the transition-dipole moments the couplings are negative and as the sum of the couplings between a given site and all other sites is negative the overall spectrum is still red-shifted as in a linear J-aggregate.

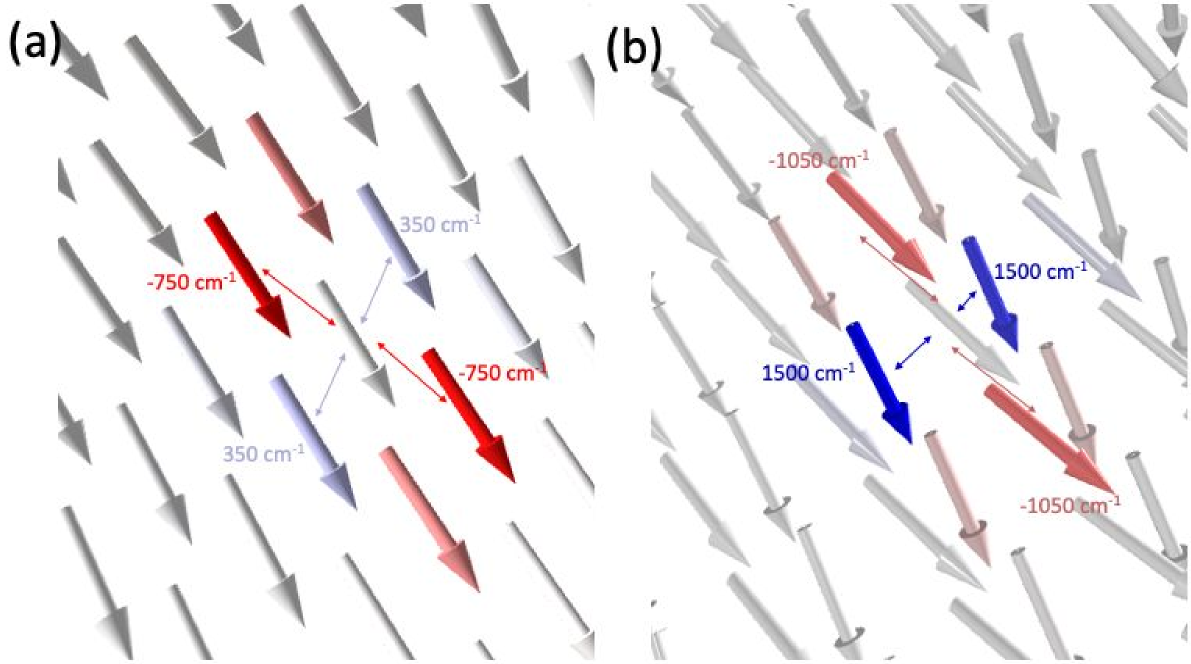

Figure S16: Panel (a) shows the signs and relative magnitudes of the couplings in the BL structure, while panel (b) shows the same for the HB structure. Blue arrows show a positive coupling with the molecule in the center, while a red color shows a negative coupling. The arrow representing the central atom is white. For the BL model the largest negative couplings are around -750 cm^{-1}, while the largest positive ones are around 350 cm^{-1}. For the HB model the largest negative couplings are around -1050 cm^{-1}, while the largest positive values are around 1500 cm^{-1}.

The exciton wavefunctions determine the exciton transition-dipoles as:

$$\vec{\mu}_{\mathrm{i}} = \sum_{m} c_{\mathrm{im}} \vec{\mu}_{m} \qquad (S10)$$

Therefore, the LDr of the PL spectra is sensitive to the wavefunction coefficients. For the BL the wavefunction coefficients are all positive for the relevant states leading to a sum of many transition-dipoles around the tube, which enhances the z-component, which is largely identical for all molecules, while the x, and y components interfere leading to a very small overall perpendicular component. In contrast for the HB structure the combination of both positive and negative wavefunction coefficients leads to a much less well-defined direction of the transition-dipole directions and a smaller overall magnitude.

In Fig. S17 transition-dipole moment scatter plots are shown for all exciton states in the two models. For the BL structure exciton states with a component parallel with the tube axis in the range between 100 and 150 Debye and a perpendicular component smaller than 10 Debye are abundant. These are the superradiant states dominating the PL. For the HB structure the largest parallel transition-dipole components are around 75 Debye. The perpendicular transition-dipole moment components of the superradiant states are generally comparable to those of the BL structure. Superradiant states for which the largest transition-dipole component is perpendicular to the tube axis are also seen in Fig. 16, however, these exciton states have higher exciton energies and do not contribute significantly to the PL as their Boltzmann weight is small.

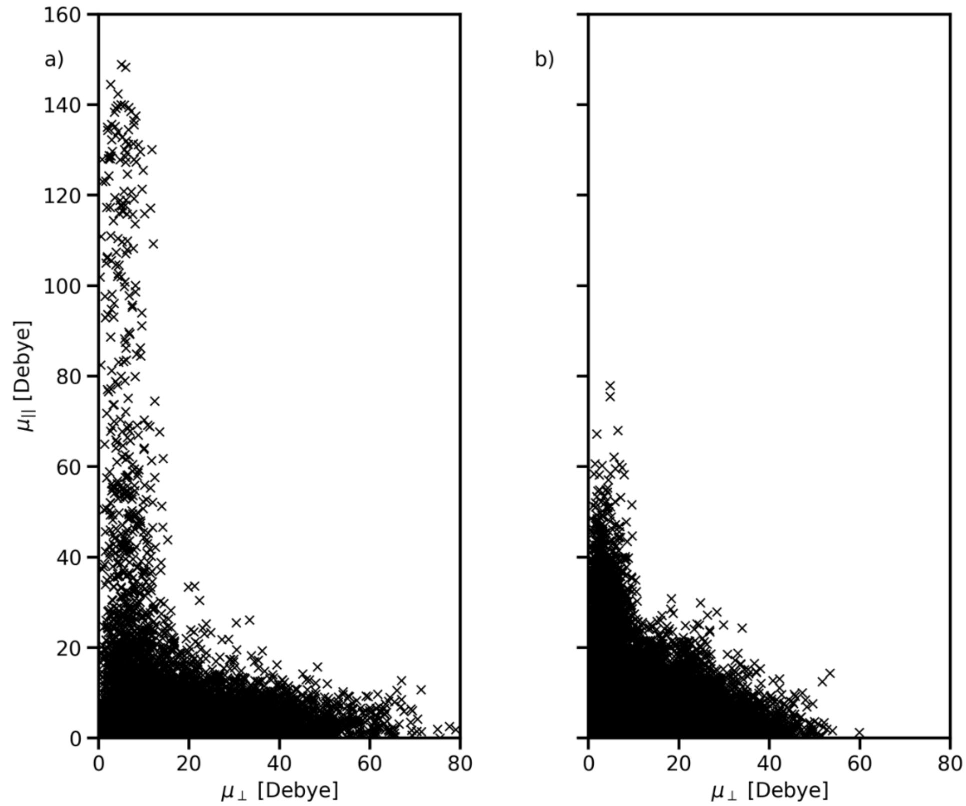

Figure S17. Scatter diagrams for the exciton transition dipole moments in the BL and HB structures shown in panel (a) and (b), respectively.

References

(1) Eisele, D. M.; Cone, C. W.; Bloemsma, E. A.; Vlaming, S. M.; Van Der Kwaak, C. G. F.; Silbey, R. J.; Bawendi, M. G.; Knoester, J.; Rabe, J. P.; Vanden Bout, D. A. Utilizing Redox-Chemistry to Elucidate the Nature of Exciton Transitions in Supramolecular Dye Nanotubes. *Nature Chem* **2012**, *4* (8), 655–662.

(2) Deshmukh, A. P.; Zheng, W.; Chuang, C.; Bailey, A. D.; Williams, J. A.; Sletten, E. M.; Egelman, E. H.; Caram, J. R. Near-Atomic-Resolution Structure of J-Aggregated Helical Light-Harvesting Nanotubes. *Nat. Chem.* **2024**, *16* (5), 800–808.

(3) Didraga, C.; Pugžlys, A.; Hania, P. R.; Von Berlepsch, H.; Duppen, K.; Knoester, J. Structure, Spectroscopy, and Microscopic Model of Tubular Carbocyanine Dye Aggregates. *J. Phys. Chem. B* **2004**, *108* (39), 14976–14985.

(4) Friedl, C.; Renger, T.; Berlepsch, H. V.; Ludwig, K.; Schmidt Am Busch, M.; Megow, J. Structure Prediction of Self-Assembled Dye Aggregates from Cryogenic Transmission Electron Microscopy, Molecular Mechanics, and Theory of Optical Spectra. *J. Phys. Chem. C* **2016**, *120* (34), 19416–19433.

(5) Fidder, H.; Knoester, J.; Wiersma, D. A. Optical Properties of Disordered Molecular Aggregates: A Numerical Study. *The Journal of Chemical Physics* **1991**, *95* (11), 7880–7890.

(6) Bondarenko, A. S.; Knoester, J.; Jansen, T. L. C. Comparison of Methods to Study Excitation Energy Transfer in Molecular Multichromophoric Systems. *Chemical Physics* **2020**, *529*, 110478.
(7) Kriete, B.; Bondarenko, A. S.; Alessandri, R.; Patmanidis, I.; Krasnikov, V. V.; Jansen, T. L. C.; Marrink, S. J.; Knoester, J.; Pshenichnikov, M. S. Molecular versus Excitonic Disorder in Individual Artificial Light-Harvesting Systems. *J. Am. Chem. Soc.* **2020**, *142* (42), 18073–18085.
(8) Bondarenko, A. S.; Patmanidis, I.; Alessandri, R.; Souza, P. C. T.; Jansen, T. L. C.; De Vries, A. H.; Marrink, S. J.; Knoester, J. Multiscale Modeling of Molecular Structure and Optical Properties of Complex Supramolecular Aggregates. *Chem. Sci.* **2020**, *11* (42), 11514–11524.
(9) Czikklely, V.; Forsterling, H. D.; Kuhn, H. Extended Dipole Model for Aggregates of Dye Molecules. *Chemical Physics Letters* **1970**, *6* (3), 207–210.
(10) Bertocchi, F.; Nizar, S.; Li, M.; Ebbesen, T. W.; Genet, C.; Painelli, A.; Cristina, Sissa. Chiroptical Properties of Cyanine Aggregates: Hierarchical Modelling from Monomers to Bundles. *Chem. Sci.* **2024**, *15*, 16103–16111.
(11) Jansen, T. L. C.; Knoester, J. Nonadiabatic Effects in the Two-Dimensional Infrared Spectra of Peptides: Application to Alanine Dipeptide. *J. Phys. Chem. B* **2006**, *110* (45), 22910–22916.
(12) Sardjan, A. S.; Westerman, F. P.; Ogilvie, J. P.; Jansen, T. L. C. Observation of Ultrafast Coherence Transfer and Degenerate States with Polarization-Controlled Two-Dimensional Electronic Spectroscopy. *J. Phys. Chem. B* **2020**, *124* (42), 9420–9427.
(13) Liang, C.; Jansen, T. L. C. An Efficient N^3 -Scaling Propagation Scheme for Simulating Two-Dimensional Infrared and Visible Spectra. *J. Chem. Theory Comput.* **2012**, *8* (5), 1706–1713.
(14) Simon, M. C. Wollaston Prism with Large Split Angle. *Appl. Opt.* **1986**, *25* (3), 369–376.
(15) Collins, T. J. ImageJ for Microscopy. *BioTechniques* **2007**, *43* (sup1), 5.
(16) Thouless, D. J. Electrons in Disordered Systems and the Theory of Localization. *Physics Reports* **1974**, *13* (3), 93–142.
(17) Jansen, T. L. C. Computational Spectroscopy of Complex Systems. *The Journal of Chemical Physics* **2021**, *155* (17), 170901.
(18) Chuang, C.; Bennett, D. I. G.; Caram, J. R.; Aspuru-Guzik, A.; Bawendi, M. G.; Cao, J. Generalized Kasha's Model: T-Dependent Spectroscopy Reveals Short-Range Structures of 2D Excitonic Systems. *Chem* **2019**, *5* (12), 3135–3150.
